# Analysis of the sorghum *RBOH* gene family and molecular mechanism of its member *SbRBOHG i*n response to aluminum stress

**DOI:** 10.1080/21645698.2026.2655482

**Published:** 2026-04-13

**Authors:** Feng Zhang, Songxian Yan, Xinlei Xu, Yingying Cheng, Junxi Yang, Haiyan Zhang, Huaijuan Gou, Lixi Deng, Moil Chu, Yanlin An, Sixia Jiang

**Affiliations:** a School of Food Engineering, Moutai Institute, Renhuai, P.R. China; b School of Brewing Engineering, Moutai Institute, Renhuai, P.R. China; c Guizhou Engineering Research Center for Comprehensive Utilization of Distillers’ Grains/School of Resources and Environmental Engineering, Moutai Institute, Renhuai, P.R. China; d School of Resources and Environmental Engineering, Moutai Institute, Renhuai, P.R. China; e Anhui Provincial Key Laboratory of the Conservation and Exploitation of Biological Resources/College of Life Sciences, Anhui Normal University, Wuhu, Anhui, China

**Keywords:** Aluminum tolerance, SbBIK1, SbRBOH gene family, SbRBOHG, sorghum

## Abstract

Respiratory Burst Oxidase Homologs (RBOHs) serve as core regulatory components in plant responses to abiotic stress, with their mediated reactive oxygen species (ROS) signaling playing a crucial role in plant adaptation to environmental challenges. This study identified 10 *SbRBOH* genes distributed across seven chromosomes in the sorghum (*Sorghum bicolor* L.) genome. Phylogenetic analysis revealed species-specific evolution within this gene family. Expression pattern analysis showed significantly higher *SbRBOHG* expression in root tissues, suggesting its potential involvement in root stress responses. Multiple sequence alignment and structural modeling revealed that SbRBOHG shares 64.79% amino acid sequence identity with *Arabidopsis* AtRBOHD, with highly conserved three-dimensional conformation. Functional studies demonstrated that heterologous overexpression of *SbRBOHG* in *Arabidopsis* enhanced plant tolerance to aluminum stress, accompanied by increased ROS accumulation in roots. Protein interaction assays (yeast two hybrid and bimolecular fluorescence complementation) further confirmed direct interaction between SbRBOHG and the kinase SbBIK1. RT-qPCR analysis also showed that *Arabidopsis* plants heterologously overexpressing the *SbRBOHG* gene activate the expression of *AtSTOP1*, a key regulator of aluminum stress, and its downstream anion channel gene, *AtALMT1*. These studies have preliminarily established the functional role of the *SbRBOHG* gene in aluminum toxicity in sorghum. The research has elucidated the physical interactions between SbBIK1 and SbRBOHG at the molecular level, providing a theoretical basis and candidate gene resources for the genetic improvement of aluminum-tolerant sorghum varieties.

## 1. Introduction

Reactive oxygen species (ROS) serve as key signaling molecules extensively involved in plant responses to biotic and abiotic stresses.^1,2^ In plant cells, ROS can be generated by various organelles, such as chloroplasts, mitochondria, and peroxisomes, through metabolic processes, or produced specifically by specialized redox enzyme systems on the plasma membrane.^1^ Among these, respiratory burst oxidase homologs (RBOHs), a class of NADPH oxidases localized to the plasma membrane, mediate localized ROS bursts and play crucial roles in plant developmental regulation and stress responses.^3^ Originally discovered in mammalian phagocytes, the catalytic core gp91phox possesses functional homologs in plants.^4^ Plant RBOHs belong to the NOX family, exhibiting distinct structural and regulatory mechanisms.^5,6^ Their proteins typically contain multiple conserved functional domains, such as the NADPH oxidase catalytic domain (NADPH-Ox), a ferric reductase-like transmembrane region, FAD and NAD binding sites,^7^ and two EF-hand domains. The latter detect calcium signals and participate in enzyme activity regulation.^8^ These structural features collectively confer RBOH with crucial functions in plant immunity and growth development.^9^ Consequently, the RBOH-mediated ROS signaling pathway represents a key mechanism for plants to respond to environmental changes and regulate their own development, with its function relying on highly conserved domains and multiple regulatory networks.

NADPH oxidases form a multi-gene family and function as core components of the ROS signaling network in plants, playing crucial roles in regulating growth, development, and stress responses.^10^ Since the initial discovery of an *RBOH* gene,^11^ homologs have been identified in a wide range of plant species, including monocots, dicots, and certain lower plants, with more than 150 members reported to date.^12,13^ The size of the RBOH family varies considerably among species: *Arabidopsis thaliana* contains 10 members (*AtRBOHA – J*),^14^ *Oryza sativa* has 9,^15^ *Nicotiana tabacum* has 14,^16^ Glycine max has 17,^17^ and *Triticum aestivum* possesses up to 46.^18^ RBOH proteins are involved in diverse physiological processes, ranging from growth and development to responses to biotic and abiotic stresses.^19–21^ In *Arabidopsis*, *AtRBOHD* and *AtRBOHF* contribute to immune responses, the regulation of ion homeostasis under salt stress, and cadmium-induced stomatal movement.^3,22–24^ *AtRBOHB* is associated with ROS production during seed germination,^25^ whereas *AtRBOHC r*egulates the apical growth of root hairs and pollen tubes.^26,27^ *AtRBOHD* and *AtRBOHF* have also been established as key elements in ABA signaling, underscoring their central role in the integration of multiple signaling pathways.^28^ Although the functions of *RBOH* genes are well characterized in *Arabidopsis*, they remain less explored in other species.^29^ Available evidence suggests that RBOH functions are largely conserved in plant immunity, microbial interactions, and responses to heavy metal stress. However, mechanistic details and pathway specificity likely differ among species, warranting further cross-species comparative and functional studies.^15,30^ In summary, the RBOH family acts as a central hub in ROS-mediated signaling and holds significant biological importance. Yet, its precise regulatory mechanisms in abiotic stress responses in non-model crops such as sorghum remain to be fully elucidated.

Of the world’s arable land, approximately 30–40% is affected by soil acidification, with the proportion rising to 70% in subtropical regions (Kochian et al. 2015). Aluminum (Al), the most abundant metal in the Earth’s crust, becomes increasingly bioavailable under acidic conditions. When soil pH falls below 5.0, Al is solubilized into the highly phytotoxic Al^3 +^ form, which impairs plant growth by inhibiting root elongation, disrupting water and nutrient uptake, reducing photosynthetic capacity, and inducing excessive accumulation of ROS (Huang et al., 2014; Kochian et al., 2015). ROS function as key early signaling molecules in plant Al^3+^ stress responses.^31^ Al exposure triggers a rapid burst of ROS in root tissues, activating downstream defense-related genes and resistance mechanisms.^31–33^ However, when ROS accumulation becomes excessive, it leads to oxidative protein modification, cellular structural damage, and metabolic dysfunction, ultimately resulting in secondary oxidative stress (Yamamoto et al. 2002^34^). To mitigate such damage, plants employ integrated antioxidant systems – comprising enzymatic and non-enzymatic components – that scavenges excess ROS and maintains redox homeostasis, thereby balancing growth and stress adaptation.^35^ A central regulator in this process is the transcription factor STOP1, whose stability is influenced by H_2_O_2_-mediated degradation pathways, modulating plant Al sensitivity.^36,37^ Studies of the Al -sensitive mutant *Atrae6* have revealed that mitochondrial H_2_O_2_ accumulation under Al stress is a critical factor in Al sensitivity, and that scavenging H_2_O_2_ can restore Al tolerance.^37^ Exogenous application of elements such as silicon and boron has also been shown to alleviate Al^3+^ toxicity by enhancing antioxidant capacity and improving ROS clearance.^35,38^ Together, these findings underscore the importance of H_2_O_2_ homeostasis in plant Al^3+^ stress adaptation.

Furthermore, plants perceive and respond to Al^3+^ stress through complex signaling mechanisms, with ROS signaling pathways attracting increasing attention. The plasma membrane-localized NADPH oxidase *AtRBOHD* serves as a key generator of ROS bursts; however, its precise function in Al stress responses remains debated. One proposed model suggests that the Al sensor ALR1/PSKR1 recruits the co-receptor AtBAK1 to activate AtRBOHD, leading to ROS production.^32^ This ROS burst is thought to inhibit degradation of the transcription factor AtSTOP1 via oxidative modification of its regulatory protein AtRAE1, thereby enhancing Al tolerance. This view is supported by observations of increased Al sensitivity and reduced AtSTOP1 accumulation in *AtrbohD* mutants. In contrast, an alternative model posits that AtRBOHD-derived H_2_O_2_ promotes the interaction between AtSTOP1 and AtRAE1, accelerating AtSTOP1 degradation and negatively regulating Al tolerance.^37^ Consistent with this, *AtrbohD* mutants exhibit enhanced Al tolerance and elevated AtSTOP1 accumulation. Further genetic evidence indicates that reducing H_2_O_2_ levels improves Al tolerance in an RAE1-dependent manner. These opposing findings underscore the complexity of ROS signaling in Al stress adaptation. The discrepancies may arise from variations in experimental conditions, genetic backgrounds, Al treatment regimens, or spatiotemporal specificity of ROS production. Future investigations should focus on elucidating how AtRBOHD-generated ROS fine-tunes the AtSTOP1–AtRAE1 module in specific cellular contexts, and whether such regulation is concentration-dependent or influenced by environmental factors.

Although previous studies have revealed the crucial role of RBOH proteins in abiotic stress responses of model plants such as *Arabidopsis* and rice, functional research on these proteins in the stress-tolerant crop sorghum remains relatively limited.^36,37^ Specifically, the precise role of certain members (e.g., SbRBOHG) in Al stress signaling pathways and their upstream regulatory mechanisms remain unclear. Furthermore, the regulatory linkages between ROS signaling and downstream key Al tolerance genes require systematic elucidation. Therefore, this study will conduct a genome-wide identification of the *SbRBOH* gene family in sorghum, analyze its evolutionary relationships and expression patterns; with a focus on elucidating the function of SbRBOHG in Al stress response. Utilizing techniques such as heterologous overexpression, protein interaction validation, and gene expression analysis, we will investigate how SbRBOHG interacts with the SbBIK1 kinase to regulate ROS bursts, thereby influencing the expression of genes associated with Al^3+^ toxicity. This research will elucidate the Al^3+^ stress signaling pathways in sorghum and reveal the molecular mechanisms underlying its Al tolerance regulation. It will provide novel targets and genetic resources for crop genetic improvement in stress resistance.

## 2. Materials and Methods

### 2.1. Plant Materials and Growth Conditions

The experimental material for this study was the wine sorghum variety “Hongyingzi” with seeds provided by Moutai Group Hongyingzi Agricultural Technology Development Co., Ltd. Seeds were germinated at 25°C and transplanted into 1/6 MS medium with an initial pH of 5.5 after emergence. This medium contained Coolaber unbuffered vitamin solution. Sorghum plants were cultivated in an artificial climate chamber set at 25°C with a photoperiod of 12 h light/12 h dark.

*Arabidopsis* wild-type Columbia ecotype (Col-0) seeds were provided by the School of Life Sciences, Anhui Normal University. Seed surface disinfection involved treatment with 75% ethanol for 3 min, followed by four rinses with double-distilled water. Seeds were then sown on 1/2 MS solid medium containing 1% sucrose and agar (Aldrich-Sigma). To break dormancy, plates were pre-chilled at 4°C in darkness for 2 d post seeding, then transferred upright and cultured at 22°C under long-day conditions (16 h light/8 h dark). Aluminum stress treatment followed previously established protocols.^39^ For Al treatment experiments, seedlings were imaged using ImageJ software on day 9 post germination to measure primary root length. A portion of seedlings were harvested for fresh weight determination to assess biomass changes. All experiments were performed in triplicate biological replicates, with data presented as mean ± standard deviation.

### 2.2. Identification and Analysis of the SbRBOH Gene Family, Chromosomal Distribution, and Replication Events

Sorghum RBOH gene family initial data were derived from protein sequences and corresponding gene annotation information in the Sorghum BTx623 reference genome provided by the Spud DB database (111.203.21.71:8000/sorghum.html). Based on BLAST homology searches (E-value threshold set at 1e^−10)^, candidate members of the RBOH gene family were preliminarily screened. Chromosomal localization analysis further validated their distribution, ultimately confirming family members and assigning systematic nomenclature. Protein molecular weight (MW) and isoelectric point (pI) parameters were predicted using ExPASy online resources (https://www.expasy.org/), while cis-acting elements in promoter regions were comprehensively analyzed via the PlantCARE database (https://bioinformatics.psb.ugent.be/webtools/plantcare/html/). Transmembrane domain prediction employed the DeepTMHMM 1.0 tool (https://dtu.biolib.com/DeepTMHMM), which utilizes deep learning algorithms to comprehensively evaluate the transmembrane topology of the SbRBOH protein family.^40^ Gene structural features, conserved motif distributions, and cis-regulatory element compositions were visualized using TBtools software. Chromosomal localization maps were constructed via the MG2C (http://mg2c.iask.in/mg2c_v2.1/) online platform based on gff annotation files. Gene duplication event analysis was performed using the MCScanX module integrated within TBtools to ensure comprehensive and reliable results.

### 2.3. Sequence Alignment, Evolutionary Analysis, and Motif Identification of RBOH Proteins

Multiple protein sequence alignments were performed using the ClustalW program within the MEGA 7 software package, with all parameters set to default values. Based on the alignment results, a phylogenetic tree was constructed using the neighbor-joining method. Evolutionary distances were calculated using a Poisson-corrected model, which is suitable for estimating genetic divergence among highly homologous sequences. The topological stability of the phylogenetic tree was validated through 1000 bootstrap replicates to assess the statistical support for each node.

To identify conserved motifs, full-length protein sequences were further scanned using the MEME Suite online analysis platform (http://meme-suite.org). Parameters were optimized as follows: maximum motif width of 50 amino acids, minimum width of 6 amino acids, and maximum number of motifs of 15. This platform employs an expectation-maximization algorithm to systematically identify statistically significant sequence motifs, providing crucial insights into the organization and evolution of protein functional domains. The aforementioned analytical methods collectively constitute a comprehensive workflow spanning sequence alignment, evolutionary analysis, and functional motif identification. This workflow establishes a reliable computational biology foundation for elucidating the functional diversification and evolutionary relationships within protein families. All analyses strictly adhere to standard bioinformatics protocols, ensuring reproducibility and comparability of results.

### 2.4. RNA Extraction and Real-Time Quantitative PCR (RT-qPCR) Analysis

Total RNA extracted from various sorghum tissue samples was processed using the TRIzol method. cDNA synthesis employed the M-MLV reverse transcriptase system with oligodT primers. Gene expression analysis was conducted on the eQ9600 real-time quantitative PCR system, with reaction conditions strictly adhering to the manufacturer’s instructions for SYBR Green I Master Mix. Specific primers for 10 members of the *SbRBOH* gene family were designed using Primer Premier 5.0 software; primer sequences are detailed in Supplementary Table S1. Each experiment included three independent biological replicates. Relative gene expression levels were calculated using the 2−ΔΔCT method and normalized against the expression of the sorghum actin gene as an internal control (-Delta Delta C(T)) method.^41^

### 2.5. Alpha Fold 2 Predicts Protein

The three-dimensional structure predictions for plant RBOH proteins were obtained from the AlphaFold Protein Structure Database (https://alphafold.ebi.ac.uk), jointly developed by Google DeepMind and EMBL-EBI, which provides structural prediction data for over 200 million proteins (Jumper et al. 2021^42^). Based on amino acid sequences, predicted structural models for sorghum SbRBOHG (UniProt ID: A0A921QXB2) and *Arabidopsis* AtRBOHD (UniProt ID: Q9FIJ0) were retrieved from the database. All structural data were generated using the AlphaFold2 algorithm, which demonstrated accuracy comparable to experimental methods in the CASP14 evaluation. Structural analysis was performed using UCSF ChimeraX 1.5 software, focusing on comparing the spatial conformations of the NADPH oxidase domain, FAD-binding domain, and six transmembrane helix regions. Model quality was assessed using predicted local distance difference test (pLDDT) scores, where the SbRBOHG model achieved a global pLDDT score of 73.25, indicating high confidence (pLDDT > 90) in the core catalytic region.

### 2.6. Construction of Fusion Expression Vectors and Subcellular Localization Analysis

Fusion expression vector For subcellular localization analysis, expression vectors pBWA(V)H2STMVΩ-egfp-SbRBOHG and pBWA(V)H2STMVΩ-egfp-SbBIK1 were constructed using molecular cloning techniques, each fused at the C-terminus to enhanced green fluorescent protein (eGFP). The specific method involved cloning the full-length coding regions of *SbRBOHG* and *SbBIK1* into the polyclonal site of the plant expression vector pBWA(V)H2STMVΩ-egfp, located upstream of the eGFP coding sequence, to form an in-frame fusion between the target gene and the reporter gene. Correctly constructed recombinant plasmids were sequenced for verification and then electro-transformed into *Agrobacterium* tumefaciens GV3101 competent cells. For transient expression experiments, *Agrobacterium* cultures containing the fusion vectors were mixed in equal proportions with cultures carrying the plasma membrane marker protein (pBWA(V)H2STMVΩ-marker). The mixture was then used to inoculate *Nicotiana benthamiana* leaves via the leaf-injection method. After inoculation, cultures were incubated under optimal conditions for 48 to 72 hours. Samples were then examined using a laser scanning confocal microscope to capture eGFP fluorescence signals. These signals were analyzed for colocalization with membrane localization markers to determine the intracellular distribution of the target protein.

### 2.7. Construction of Bimolecular Fluorescent Complementary Carriers and Detection of Protein Interactions

To validate protein-protein interactions, a bimolecular fluorescent complementation (BiFC) detection system was established. First, the dual-fluorescence complementation expression frameworks pSPYNE(R) and pSPYCE(M) were cloned into the plant binary vector pCAMBIA1300, yielding recombinant vectors p1300-SPYNE(R) and p1300-SPYCE(M). Following restriction enzyme digestion, ligation, transformation, and sequencing verification, candidate interacting genes SbRBOHG and SbBIK1 were inserted into the respective vectors to construct BiFC expression constructs: p1300-SPYNE(R)-SbBIK1/p1300-SPYCE(M)-SbRBOHG was used to detect heterologous interaction, with p1300-SPYNE(R)-SbBIK1/p1300-SPYCE(M)-SbBIK1 serving as the homologous interaction control. Each BiFC vector pair was electro-transformed into *Agrobacterium* tumefaciens GV3101. The resulting co-transformed strains were mixed and used to co-inoculate *Nicotiana benthamiana* leaves via the co-inoculation method. At designated post-inoculation time points, the appearance of yellow fluorescent protein complementation signals was observed and recorded using a laser scanning confocal microscope to determine whether direct interactions occurred between the target proteins within plant cells. Empty vectors and known interacting protein pairs were included as negative and positive controls, respectively.

### 2.8. Cloning of the Full-Length CDS Sequence of the SbRBOHG and SbBik1 Genes

From “Hongyingzi” sorghum (total RNA extracted from root tissue using a kit purchased from Vazyme Biotech Co., Ltd. Using this RNA as a template, the first-strand cDNA was synthesized with M-MLV reverse transcriptase. Using this cDNA as a template, PCR amplification was performed with specific primers SbRBOHG-F1/SbRBOHG-R1 and SbBIK1-F1/SbBIK1-R1 (sequences detailed in Supplementary Table S1). The reaction program was as follows: 5 min pre-denaturation at 95°C; followed by 35 cycles (95°C for 30 s, 58–60°C for 30 s, 72°C for 1 min); and a final extension at 72°C for 5 min. Amplified products were separated by electrophoresis on a 1% (w/v) agarose gel (containing GelRed nucleic acid dye) at 120 V for 30 min. Target bands were purified using a DNA gel extraction kit, and concentrations and purity of the *SbRBOHG* and *SbBIK1* fragments were determined using a METTLER TOLEDO UV5_Nano_ micro-spectrophotometer. Purified PCR products were A-tailed with Taq DNA polymerase at 72°C for 15 min. Subsequently, the products were ligated with pMD19-T vector overnight at 16°C. The ligation products were transformed into E. coli DH5α competent cells via heat shock, spread onto LB solid medium containing ampicillin (100 μg/mL), X-gal, and IPTG, and incubated at 37°C for 12–16 h. White single colonies were picked for colony PCR verification. Positive clones were submitted for sequencing. The obtained sequences were compared and corrected. After confirmation of accuracy, they were used for subsequent experiments.

### 2.9. The Creation and Characterization of SbRBOHG Overexpressing Arabidopsis Materials

The complete coding sequence (CDS) of *SbRBOHG* was amplified by PCR using cDNA from the root tissue of red-tasseled sorghum as a template. After double digestion with NcoI/SpeI, this fragment was cloned into the corresponding site of the plant expression vector pCAMBIA1302, yielding the recombinant plasmid pCAMBIA1302-35S-SbRBOHG driven by the 35S constitutive promoter. Following sequencing verification, the recombinant vector was electro-transformed into *Agrobacterium* tumefaciens strain GV3101. The above *Agrobacterium*-transformed *Arabidopsis* WT (Col-0) plants were transformed using the flower-dipping method (Clough and Bent 1998). Transformed seeds were collected, surface-sterilized, and evenly spread on petri dishes containing 1/2 MS solid medium supplemented with 35 μg•mL^− 1^ kanamycin. Seeds were vernalized at 4°C for 4 d, then transferred to a growth chamber (25°C, 16 h light/8 h dark) for 8 d. Surviving seedlings were transplanted into nutrient soil for further cultivation. T1 transgenic positive plants were screened via genomic PCR using designed specific primers (Table S1). Through consecutive self-pollination and purification, three independent homozygous T3 generation overexpression lines were obtained. RT-PCR was used to determine the expression levels of *SbRBOHG* in each line, and the line with the highest expression (OE-*SbRBOHG#5*) was selected for subsequent Al stress tolerance analysis (Figure S5).

### 2.10. Hematoxylin and Morin Fluorescent Staining Analysis of Root Aluminum Content

Plates containing 1/2 MS basal medium were prepared, with the experimental group supplemented with 300 μM aluminum chloride and the control group without Al^3+^ supplementation. Following sterilization, all media were poured into sterile Petri dishes and allowed to solidify. For staining analysis, hematoxylin solution (50 mL) was prepared by dissolving 0.1 g hematoxylin and 0.01 g potassium iodate in ultrapure water with constant stirring. After complete dissolution, 50 μL of 1 M NaOH solution was added, with the solution prepared fresh for immediate use. Morin staining solution was prepared by first dissolving 6.0448 g Morin in 5 mL methanol to generate a 4 mM stock solution, which was stored at 4°C. For working solution preparation, 1 mL of stock solution was diluted with 39

WT and overexpressing *Arabidopsis* seedlings grown vertically for 5 days under controlled conditions were selected for experimentation. Under sterile conditions in a laminar flow hood, plants with uniform root length and vigorous growth were transferred to control or Al^3+^ treated plates using sterile toothpicks. All plates were sealed with sterile breathable film and maintained in the growth chamber for 6 hours. Following treatment, seedlings were harvested and rinsed three times with ultrapure water. The cleaned seedlings were immersed in 2 mL of staining solution in 24 well plates, covered with lids, wrapped in Al^3+^ foil for light protection, and stained at 22°C for 30 min. After staining, samples were washed three times with ultrapure water, mounted on glass slides, and examined under a microscope for image acquisition. All treatments included three independent biological replicates with appropriate controls to ensure experimental reliability.

### 2.11. Statistical Analysis

All experiments included at least three independent biological replicates. Data are expressed as mean ± standard deviation (SD), and intergroup comparisons were performed using one-way analysis of variance (ANOVA). When ANOVA indicated significant differences, post hoc multiple comparisons were further conducted using Fisher’s least significant difference (LSD) test. The significance level was set at α = 0.05, meaning p values < 0.05 were considered statistically significant. Statistical analyses were performed using SPSS 22.0 software.

## 3. Results

### 3.1. Comparative Genomic Analysis of the Sorghum RBOH Gene Family Reveals Uneven Chromosomal Distribution and Localized Amplification Characteristics

Previous studies have demonstrated significant variation in the number of *RBOH* gene family members across different plant species. The model plant *Arabidopsis thaliana* contains 10 *RBOH* family members,^14^ while *Solanaceae* crops such as tomato (*Solanum lycopersicum*) and eggplant (*Solanum melongena*) each possess 8 members.^43,44^ Based on published sorghum genome data, phylogenetic analysis and conserved domain identification identified 10 genes encoding the classic *RBOH* domain in the sorghum genome. These were named *SbRBOHA* to *SbRBOHJ* according to their chromosomal locations (Figure S1 and Table S2).

**Figure 1.**
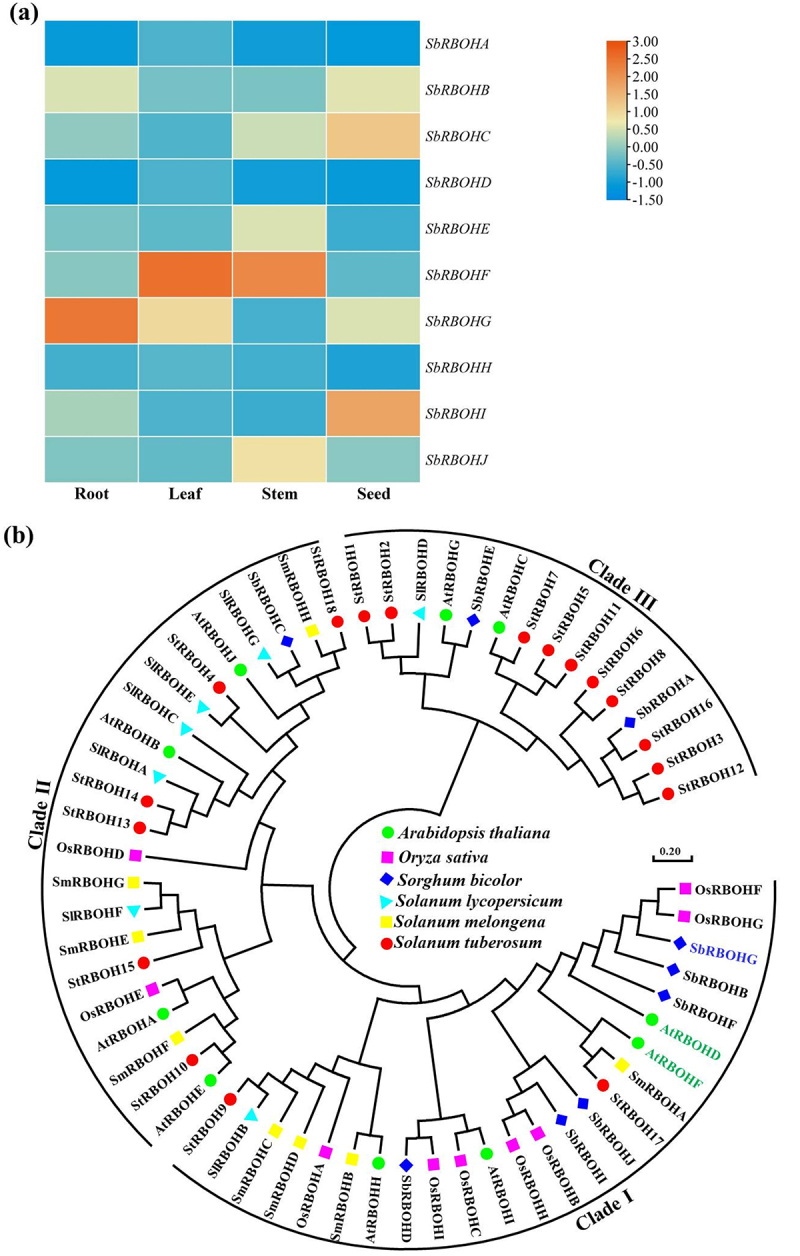
Expression patterns and phylogenetic analysis of the sorghum *SbRBOH* gene family. (a) Expression heatmap of 10 sorghum *SbRBOH* genes in root, leaf, stem, and seed tissues. Color intensity indicates gene expression levels, with blue representing low expression and orange indicating high expression. Results reveal distinct tissue-specific expression patterns among different members, with some genes showing high-specific expression in roots. (b) Phylogenetic tree of RBOH proteins from sorghum, *Arabidopsis*, rice, tomato, eggplant, and potato. Phylogenetic analysis grouped RBOH proteins into three major clades (i, ii, iii), revealing evolutionary relationships and taxonomic distribution across species. Sorghum SbRBOH members were distributed across these clades, suggesting potential functional diversification during evolution.

Chromosomal localization analysis revealed uneven distribution of *SbRBOH* family members across the sorghum genome. The 10 genes are distributed across 7 chromosomes, with chromosome 3 containing 4 members (*SbRBOHC*, *SbRBOHD*, *SbRBOHE*, and *SbRBOHF*), while the remaining chromosomes each contain only one member (Figure 1). These genes are relatively dispersed on their respective chromosomes, failing to form distinct gene clusters. Notably, the 4 genes on chromosome 3 occupy distinct physical positions, exhibiting a pattern of spaced distribution. Genes are distributed with similar proportions on both positive and negative strands, indicating no significant preference for transcription direction. This uneven chromosomal distribution pattern suggests that the *RBOH* gene family may have undergone localized expansion events during sorghum evolution. The dense clustering of genes on chromosome 3 warrants particular attention, potentially reflecting functional differentiation or synergistic regulatory relationships among these genes. These findings provide crucial genomic insights for elucidating the functional specificity of the *RBOH* gene family in sorghum growth, development, and stress responses.

### 3.2. Phylogenetic and Structural Analysis Reveals Evolutionary Differentiation of the SbRBOH Gene Family

To elucidate the evolutionary and structural characteristics of the sorghum *RBOH* gene family, phylogenetic, conserved domain, and gene structure analyses were conducted. Phylogenetic analysis revealed that the 10 *SbRBOH* members can be grouped into three major branches (Group I – III): Group I comprises 7 members, Group II contains 2 members, and Group III includes only 1 member, suggesting significant diversification within this family during evolution (Figure S2). Analysis of conserved domains revealed that all SbRBOH proteins possess the typical NADPH oxidase core domain, including an N-terminal transmembrane domain, an intracellular FAD-binding domain, and a NADPH-binding domain, indicating high catalytic functional conservation. However, variations in the number and arrangement of N-terminal regulatory regions (e.g., EF-hand calcium-binding domains) among group members suggest potential functional specialization in calcium signaling perception and activation mechanisms. Gene structure analysis further revealed that members within the same evolutionary group share similar exon-intron compositions, while significant structural differences exist between groups. This result supports the phylogenetic grouping and indicates that the family likely achieved functional diversification through gene duplication followed by structural variation.

Additionally, analysis of *SbRBOH* gene family chromosomal distribution identified four pairs of duplication events, including tandem duplications on different chromosomes (e.g., *SbRBOHE* and *SbRBOHJ* on Chr03 and Chr09) and segmental duplications between different chromosomes (e.g., *SbRBOHB* on Chr02 and *SbRBOHH* on Chr07) (Figure S3). These duplication pairs exhibit uneven distribution across seven chromosomes, with duplication-related members present on Chr02, Chr03, Chr07, and Chr09, while no significant duplication events were detected on Chr01, Chr05, and Chr08. This distribution pattern suggests that the expansion of the *SbRBOH* gene family likely relies primarily on local tandem duplication and genomic segmental duplication. The chromosomal localization differences resulting from these duplication events may further influence their expression regulation patterns and functional specialization, providing an evolutionary genomics basis for understanding the functional specificity of this family in stress responses.

### 3.3. Expression Profiling and Phylogenetic Analysis Suggest SbRBOHG as a Key Candidate Gene in Sorghum Root Stress Response

A genome-wide identification of the sorghum respiratory burst oxidase (*SbRBOH*) gene family was performed to characterize its members and analyze their expression patterns and evolutionary relationships. Bioinformatic analysis identified 10 *SbRBOH* genes (SbRBOHA – J) in the sorghum genome. Expression heatmap analysis across different tissues (roots, leaves, stems, seeds) revealed distinct tissue-specific expression profiles among the family members (Figure 1(a)). Genes such as *SbRBOHA* and *SbRBOHB* were highly expressed in vegetative organs (roots, leaves, stems), while *SbRBOHC* and *SbRBOHD* showed preferential expression in seeds, suggesting specialized roles in seed development or germination. Notably, *SbRBOHG* was highly and specifically expressed in roots, implying a potential function in root biology or stress adaptation.

To trace the evolutionary history of the SbRBOH family, a phylogenetic tree was constructed using RBOH protein sequences from sorghum, *Arabidopsis thaliana*, *Oryza sativa*, and other species (Figure 1(b)). The analysis showed that all SbRBOH proteins cluster within established plant RBOH phylogenetic groups. Sorghum RBOHs were more closely related to those from rice, a monocot, consistent with evolutionary divergence. Importantly, SbRBOHG grouped in the same clade as AtRBOHD and AtRBOHE from *Arabidopsis*, both implicated in stress signaling. This phylogenetic association supports the hypothesis that SbRBOHG may have a conserved role in environmental stress responses. In summary, the *SbRBOH* gene family exhibits both tissue-specific expression patterns and evolutionary relationships indicative of functional diversification and conservation. The root-predominant expression of *SbRBOHG* and its phylogenetic proximity to known stress-related RBOH genes provide important insights for further functional studies on its role in sorghum root stress physiology.

### 3.4. Multi-Species RBOH Protein 3D Structure Prediction Based on AlphaFold2 Reveals High Structural Similarity Between SbRBOHG and AtRBOHD

**Figure 2.**
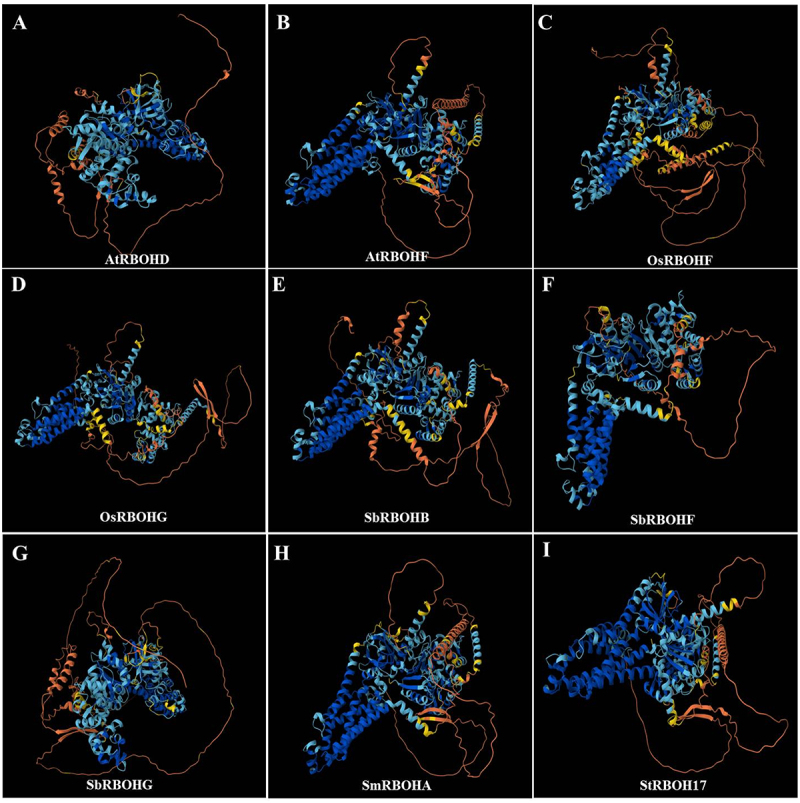
Comparative analysis of three-dimensional structures and conservation of RBOH proteins across plant species. AlphaFold2 software was employed to predict the three-dimensional structures of multiple RBOH homologs from sorghum (*Sorghum bicolor*), *Arabidopsis thaliana*, rice (*Oryza sativa*), eggplant (*Solanum melongena*), and potato (*Solanum tuberosum*), including AtRBOHD, AtRBOHF, OsRBOHF, OsRBOHG, SbRBOHB, SbRBOHF, SbRBOHG, SmRBOHA, and StRBOH17. All proteins exhibited the characteristic NADPH oxidase Fold, comprising a conserved NADPH-binding domain, FAD-binding domain, and transmembrane helix structure. Structural alignment revealed that despite differing species origins, RBOH proteins showed high overall topological conservation. However, conformational variations were observed in the intracellular loop region and N-terminal regulatory domain, potentially influencing their specific interactions with regulatory factors such as kinases and calcium ions. Notably, SbRBOHG (Figure a) and AtRBOHD (Figure G) exhibit high similarity in their three-dimensional structures, suggesting potential functional conservation between the two.

The three-dimensional structures of RBOH proteins from sorghum, *Arabidopsis*, rice, potato, and tomato were predicted using AlphaFold 2 to assess structural conservation and variation among species. All examined RBOHs – including AtRBOHD, AtRBOHF, OsRBOHF, OsRBOHG, SbRBOHB, SbRBOHF, SbRBOHG, SmRBOHA, and StRBOH17 displayed a conserved topological architecture featuring continuous α-helices and β-sheets that form essential NADPH-binding, FAD-binding, and transmembrane domains (Figure 2). While the overall fold was highly consistent, variations were observed in the conformation and length of intracellular loops and certain surface helices, which may influence interactions with regulatory proteins such as kinases or small GTPases.

Among the three sorghum RBOH proteins analyzed, SbRBOHB, SbRBOHF, and SbRBOHG shared a similar structural scaffold. However, SbRBOHG displayed a more extended N-terminal region, which typically contains EF-hand motifs involved in calcium sensing, suggesting distinct mechanisms for calcium-dependent activation. Structural alignment revealed that SbRBOHG closely resembles *Arabidopsis* AtRBOHD and rice OsRBOHG, particularly within the cytoplasmic catalytic core responsible for ROS production. This structural conservation supports a potential functional analogy, implicating SbRBOHG in root development and stress signaling. In contrast, SbRBOHB and SbRBOHF showed more pronounced conformational differences in surface loop regions relative to orthologs from other species, indicating possible functional divergence. In summary, plant RBOH proteins maintain a highly conserved structural core while exhibiting local variations that may underlie functional specificity. The structural resemblance of SbRBOHG to well-characterized ROS-generating enzymes such as AtRBOHD provides a basis for further mechanistic studies using structural modeling and site-directed mutagenesis.

### 3.5. SbRBOHG Localizes to the Plasma Membrane and Exhibits Significant Expression in the Root Tip Region

**Figure 3.**
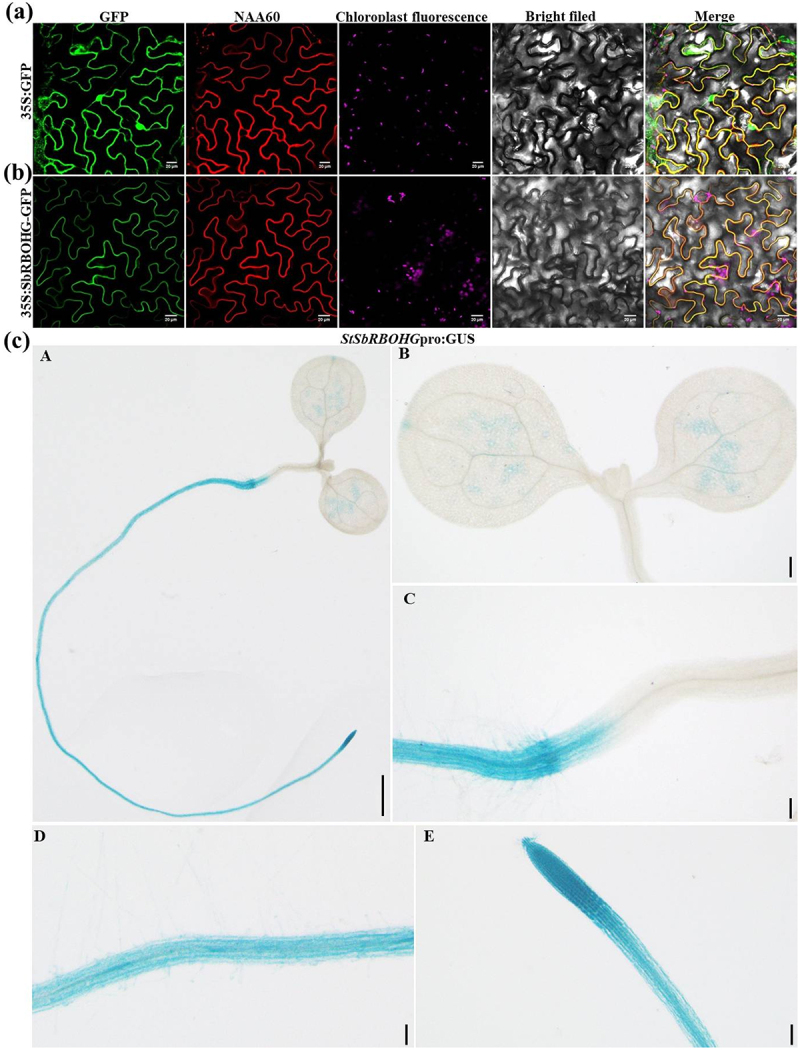
Subcellular localization and tissue-specific expression analysis of *SbRBOHG*. (a-b) Subcellular localization analysis of SbRBOHG. (a) 35S:GFP empty vector control shows GFP signal distributed in the cytoplasm and nucleus; (b) 35S:SbRBOHG-GFP fusion protein expressed in tobacco epidermal cells, showing localization to the plasma membrane. Images from left to right: GFP green fluorescence signal, NAA60 (positive control), chloroplast autofluorescence (purple), brightfield image, and multi-channel merged image. Scale bar = 20 μm; (c) Histochemical staining analysis of St*SbRBOHG*pro:GUS heterotransgenic Arabidopsis plants. GUS staining reveals high promoter activity in root apical meristems (C), elongation zones (d), and root hair regions (d), with weaker expression in leaves (b) and stems (e). These results indicate SbRBOHG is specifically highly expressed in roots, suggesting potential involvement in root growth, development, and stress response processes.

For analyzing the subcellular localization characteristics and tissue expression patterns of *SbRBOHG*, both fluorescent protein fusion localization and promoter-driven GUS histochemical staining analyses were conducted. Transient expression of the 35S: *SbRBOHG*-GFP fusion protein in *N. benthamiana* epidermal cells revealed confocal microscopy observations (Figure 3a) showing that in the empty vector GFP control group, green fluorescence signals were uniformly distributed throughout the cytoplasm and nucleus. In contrast, cells expressing SbRBOHG-GFP exhibited distinct fluorescence signals localized to the cell periphery, aligning with the morphology of the plasma membrane and distinct from chloroplast auto-fluorescence regions. This indicates that SbRBOHG is a plasma membrane-localized protein (Figure 3b). This result aligns with the typical characteristics of RBOH family proteins, suggesting it may participate in extracellular ROS production and trans-membrane signaling through its plasma membrane localization.

Furthermore, the expression specificity of *SbRBOHG* was analyzed using a promoter-driven GUS reporter system. Histochemical staining revealed (Figure 3(c)) that GUS activity was significantly enriched in root tissues of the transgenic *Arabidopsis* plants, with intense blue precipitation observed particularly in the root apical meristem, elongation zone, and root hair regions (Figure 3(C,D)). This indicates high transcriptional activity of the *SbRBOHG* promoter in root tip and root hair cells. In contrast, leaves (Figure 3(C,B)) and stems (Figure 3(C,E)) exhibited only faint staining, indicating low expression levels in above-ground tissues. This expression pattern aligns with *SbRBOHG*‘s root specific high expression in transcriptomic data, further supporting its potential crucial role in root growth, development, and stress responses. These findings demonstrate that SbRBOHG localizes to the plasma membrane at the protein level and exhibits root-specific expression patterns at the tissue level, with particularly active expression in root tips and root hair regions. This provides cellular and histological evidence for its involvement in root-specific physiological processes, such as Al stress response.

### 3.6. Heterologous Overexpression of the SbRBOHG Gene Reduces Root Tip Aluminum Accumulation and Confers Aluminum Tolerance to Arabidopsis

**Figure 4.**
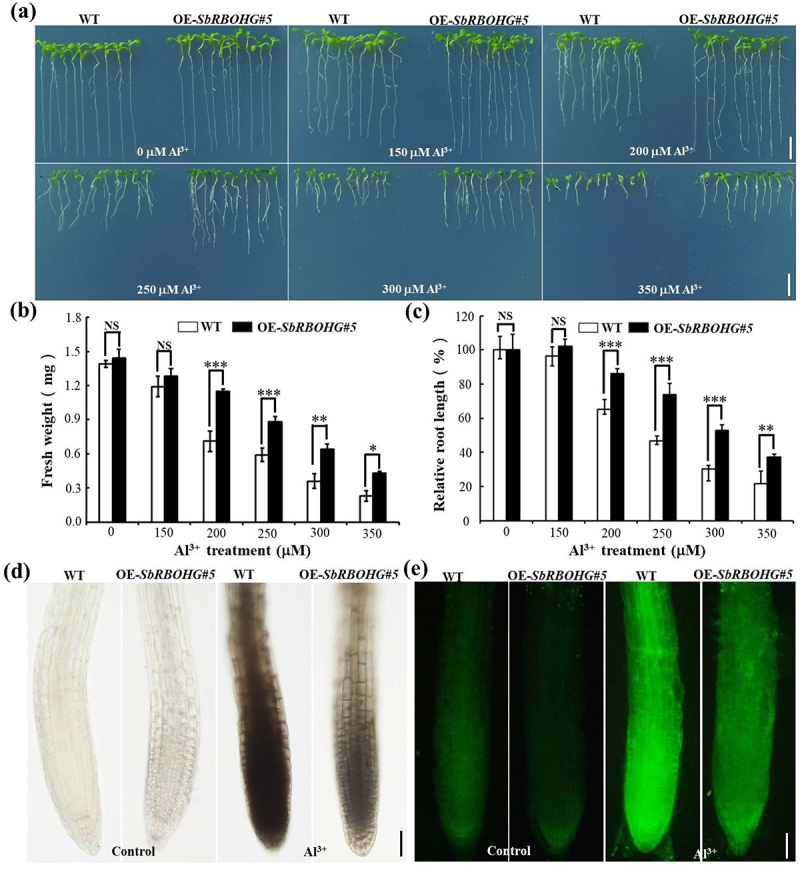
Phenotypic and physiological analysis of *SbRBOHG* heterologous overexpression enhancing aluminum tolerance in *Arabidopsis*. (a – c) Effects of *SbRBOHG* heterologous overexpression on *Arabidopsis* Al^3+^ tolerance. (a) Phenotypic comparison of wt and *SbRBOHG* overexpressing line (OE-*SbRBOHG#5*) under varying Al^3+^ concentrations (0–350 μM). As Al^3 +^ concentration increased, wt plant growth was progressively inhibited, whereas the overexpressing line maintained robust growth. (b) Fresh weight statistics. (c) Relative root length statistics. Data indicate that under 200–350 µM Al^3+^ stress, fresh weight and root length of the overexpressing line significantly exceeded those of wt (*p* < .05, *p < .01); (d) Hematoxylin staining analysis of root tip Al^3+^ accumulation. wt root tips exhibited distinct dark precipitates after Al treatment, indicating substantial Al^3+^ accumulation; in contrast, the OE-*SbRBOHG#5* transgenic line showed significantly reduced staining, suggesting SbRBOHG may reduce aluminum ion accumulation in root tips; (e) Morin fluorescence staining assay. Under Al^3+^ stress, the root tips of OE-SbRBOHG#5 plants exhibited stronger fluorescence signal intensity compared to wt, further confirming that overexpression of the SbRBOHG gene reduces aluminum content in the root tips.

To investigate the role of *SbRBOHG* in Al^3+^ stress tolerance, we generated transgenic *Arabidopsis thaliana* lines overexpressing *SbRBOHG* under the control of the 35S promoter (OE-*SbRBOHG#5*) via *Agrobacterium*-mediated transformation. When subjected to a gradient of Al^3+^ concentrations (0–350 μM), wild-type plants displayed characteristic Al^3+^ toxicity symptoms, including suppressed root elongation and reduced biomass (Figure 4(a)). In contrast, the OE-*SbRBOHG#5* line maintained vigorous growth at Al^3+^ levels ≥ 200 μM. The overexpression line showed a 20–40% higher fresh weight retention rate compared to wild-type plants (Figure 4(b)). Relative root length measurements further indicated that root elongation inhibition was significantly milder in the transgenic line under 200–350 µM Al^3+^ treatment (Figure 4(c)). No notable differences were observed between genotypes at Al^3+^ concentrations ≤ 150 µM, suggesting that *SbRBOHG* may function predominantly under high-stress conditions. These findings demonstrate that *SbRBOHG* overexpression enhances Al^3+^ tolerance in *Arabidopsis* by sustaining root growth and biomass accumulation.

To explore the physiological basis of *SbRBOHG* mediated Al^3+^ tolerance, we examined aluminum distribution in root tips using hematoxylin and morin staining (Figure 4(d,e)). Hematoxylin staining revealed pronounced dark-brown deposits in the root meristem of wild-type plants under Al^3+^ stress, indicating substantial Al^3+^ accumulation. In *SbRBOHG* overexpressing plants, the stained region was approximately 30% shorter and staining intensity was markedly weaker (Figure 4(d)). Morin staining corroborated these results, with fluorescence signal intensity in overexpression lines reduced to 45% of that in wild-type roots, reflecting less aluminum – dye complex formation (Figure 4(e)). Collectively, these data suggest that *SbRBOHG* may enhance Al^3+^ tolerance through a dual mechanism: limiting Al^3+^ transport and accumulation in the root apical meristem, and promoting Al^3+^ immobilization in the cell wall or activating efflux pathways. This regulatory influence helps preserve root tip function and improves overall Al^3+^ tolerance, providing new insight into the physiological role of *SbRBOHG* in plant responses to Al^3+^ stress.

### 3.7. SbRBOHG Positively Regulates Aluminum Stress-Induced Reactive Oxygen Species Bursts by Enhancing NADPH Oxidase Activity

**Figure 5.**
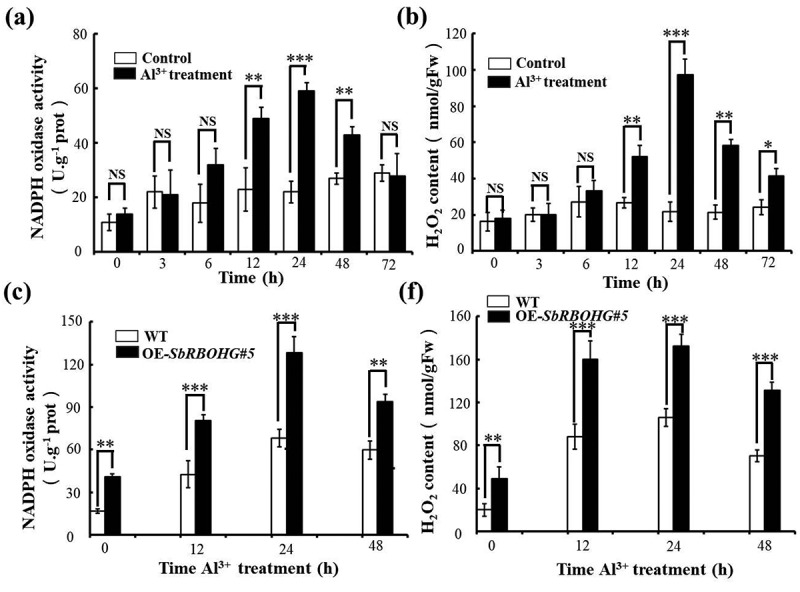
The Effects of Al^3+^ stress and *SbRBOHG* overexpression on NADPH oxidase activity and H_2_O_2_ accumulation in Arabidopsis roots. (a) Dynamic changes in NADPH oxidase activity in wt *Arabidopsis* roots under Al^3+^ stress (+Al^3+^) and control (-Al^3+^) conditions from 0 to 72 hours. Enzyme activity significantly increased after 12 h of Al^3+^ treatment (*p* < .05, *p < .01), indicating Al^3+^ stress activates NADPH oxidase; (b) Changes in H_2_O_2_ content over the same time course. H_2_O_2_ accumulation remained consistently higher in Al^3+^ treated groups than controls, further confirming Al^3+^ stress induces reactive oxygen species bursts; (c) Comparison of NADPH oxidase activity between wt and *SbRBOHG*-overexpressing line (OE-*SbRBOHG#5*) under Al^3+^ stress. Enzyme activity in the overexpressing line was significantly higher than wt from 12 to 48 hours (*p < .001), indicating *SbRBOHG* participates in regulating Al^3+^ induced oxidase activation; (d) Promoting effect of *SbRBOHG* overexpression on H_2_O_2_ accumulation. H_2_O_2_ content in OE-*SbRBOHG#5* plants consistently exceeded that of wt under Al^3+^ treatment, with differences becoming more pronounced over time. This suggests *SbRBOHG* amplifies Al^3+^ stress signals through positive feedback by enhancing ROS bursts. Error bars indicate mean ± standard deviation (*n* = 6).

In order to elucidate the temporal regulatory role of SbRBOHG in Al^3+^ stress response, dynamic analysis was conducted on NADPH oxidase activity and H_2_O_2_ accumulation in Arabidopsis root tissues. 12 hours after Al^3+^ stress application, NADPH oxidase activity in wild-type plants significantly increased, peaking at 24 h with activity levels approximately 1.5-fold higher than the control group (Figure 5(a)). H_2_O_2_ content rose synchronously 12 h after Al^3+^ treatment, peaking at 24 h, with a trend consistent with enzyme activity dynamics (Figure 5(b)). This indicates that early Al^3+^ stress activates the NADPH oxidase-mediated reactive oxygen species burst pathway. Comparative analysis revealed that roots of the *SbRBOHG* overexpressing line (OE-*SbRBOHG#5*) exhibited significantly higher NADPH oxidase activity than the wild-type at 12 h post-aluminum treatment, with this difference persisting until 48 h (Figure 5(c)). Correspondingly, H_2_O_2_ accumulation in the overexpressing line exceeded that of the WT at all-time points, with the most pronounced difference observed at 24 h, showing a 43% increase (Figure 5(d)). These findings demonstrate that SbRBOHG participates in the early ROS burst during Al^3+^ stress by enhancing NADPH oxidase activity, with its expression level directly influencing ROS production intensity. This discovery provides experimental evidence for elucidating the core function of SbRBOHG in aluminum signaling pathways and offers a new perspective on understanding redox regulatory mechanisms in plant Al^3+^ stress responses.

### 3.8. SbBik1 and SbRBOHG Amplify AtStop1-AtAlmt1 Expression Through H_2_O_2_ Mediated Signaling Cascades to Enhance Sorghum Al Tolerance

**Figure 6.**
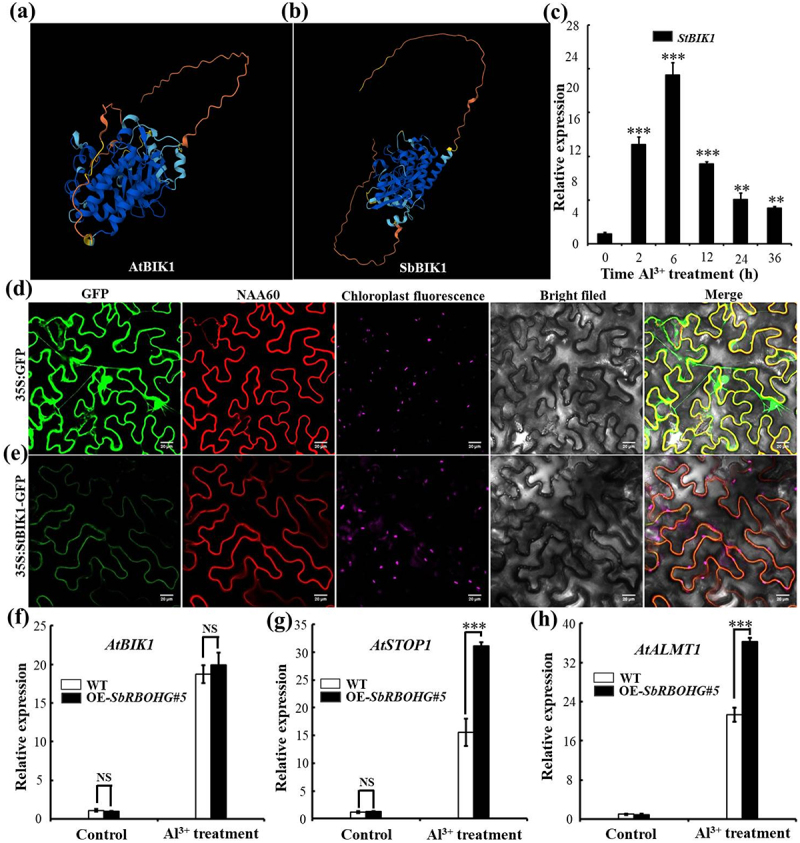
Protein structure, expression pattern, subcellular localization, and functional analysis of SbBIK1 in the aluminum stress signaling pathway. (a, b) Predicted three-dimensional protein structure models of AtBIK1 (a) and SbBIK1 (b). Structural comparison reveals highly conserved folding patterns in the kinase domains of both proteins, suggesting potential functional similarity in kinase activity. (c) Transcriptional expression of *SbBIK1* under Al^3+^ stress. Relative expression of SbBIK1 significantly increased within 2 h after Al^3+^ treatment and peaked at 6 h. (d, e) Subcellular localization of SbBIK1. (d) GFP signal from the 35S: GFP empty vector control was uniformly distributed in the cytoplasm and nucleus. (e) Fluorescence signal of 35S:SbBIK1-GFP fusion protein specifically localized to the plasma membrane, clearly visible after overlaying with positive control NAA60 fluorescence (red) and bright field images, confirming SbBIK1 as a membrane-associated protein. Scale bar = 20 μm. (f – h) Effects of SbRBOHG overexpression on Al^3+^ stress related gene expression. Relative expression levels of (f) *StBIK1*, (g) *StSTOP1*, and (h) *StALMT1* in wild-type (wt) and overexpressing line (OE-SbRBOHG#5) under control (-Al^3+^) and Al^3+^ treated (+al) conditions. Al^3+^ stress significantly induces expression of all three genes in wt; in OE-*SbRBOHG#5*, Al^3+^ induced expression levels of *StSTOP1* and *StALMT1* are further significantly up-regulated. Error bars indicate mean ± standard deviation (*n* = 3). Asterisks indicate significant intergroup differences determined by t-tests (*p* < .05, *p* < .01, *p* < .001).

In plants, BIK1 directly phosphorylates RBOHD, thereby triggering immune responses such as ROS bursts and cytoplasmic calcium influx.^45,46^ However, the function of the BIK1-RBOHD signaling module under abiotic stress remains unclear. To investigate the molecular mechanism of sorghum SbRBOHG in responding to Al^3+^ stress, this study systematically analyzed the SbBIK1 kinase and its downstream signaling events potentially involved in this process. Sequence and structural comparisons revealed high protein structural similarity between sorghum SbBIK1 and *Arabidopsis* AtBIK1, suggesting potential functional conservation (Figure 6(a,b)). Expression analysis under Al^3+^ stress revealed that *SbBIK1* transcription peaked at 6 h and then gradually declined (Figure 6(c)), indicating this gene may function as an early response factor in Al^3+^ stress signaling. Subcellular localization experiments further demonstrated that the SbBIK1-GFP fusion protein primarily localized to the plasma membrane (Figure 6(d,e)), suggesting it may act through membrane-associated signaling pathways.

To further elucidate the regulatory role of ROS in Al stress responses, this study examined expression changes of key signaling genes in *SbRBOHG#5* overexpressing plants. Under Al^3+^ stress, the expression levels of *StSTOP1* and *StALMT1* were significantly higher in the overexpressing lines compared to wild-type plants, while *SbBIK1* expression showed no obvious changes (Figure 6(f-h)). These results suggest that SbRBOHG may enhance sorghum Al tolerance by mediating H_2_O_2_ accumulation, thereby activating the SbSTOP1-SbALMT1 signaling pathway. In summary, this study preliminarily reveals a potential signaling pathway involving SbBIK1 and SbRBOHG in sorghum’s Al^3+^ stress response: Al^3+^ stress induces early expression of SbBIK1, which activates SbRBOHG. This promotes H_2_O_2_ accumulation and synergistically regulates the plant’s adaptive response to Al^3+^ toxicity by up-regulating key genes such as SbSTOP1 and SbALMT1.

### 3.9. The Molecular Basis of the Al^3+^ Stress Signaling Pathway Formed by SbBik1 and SbRBOHG Interacting at the Plasma Membrane

**Figure 7.**
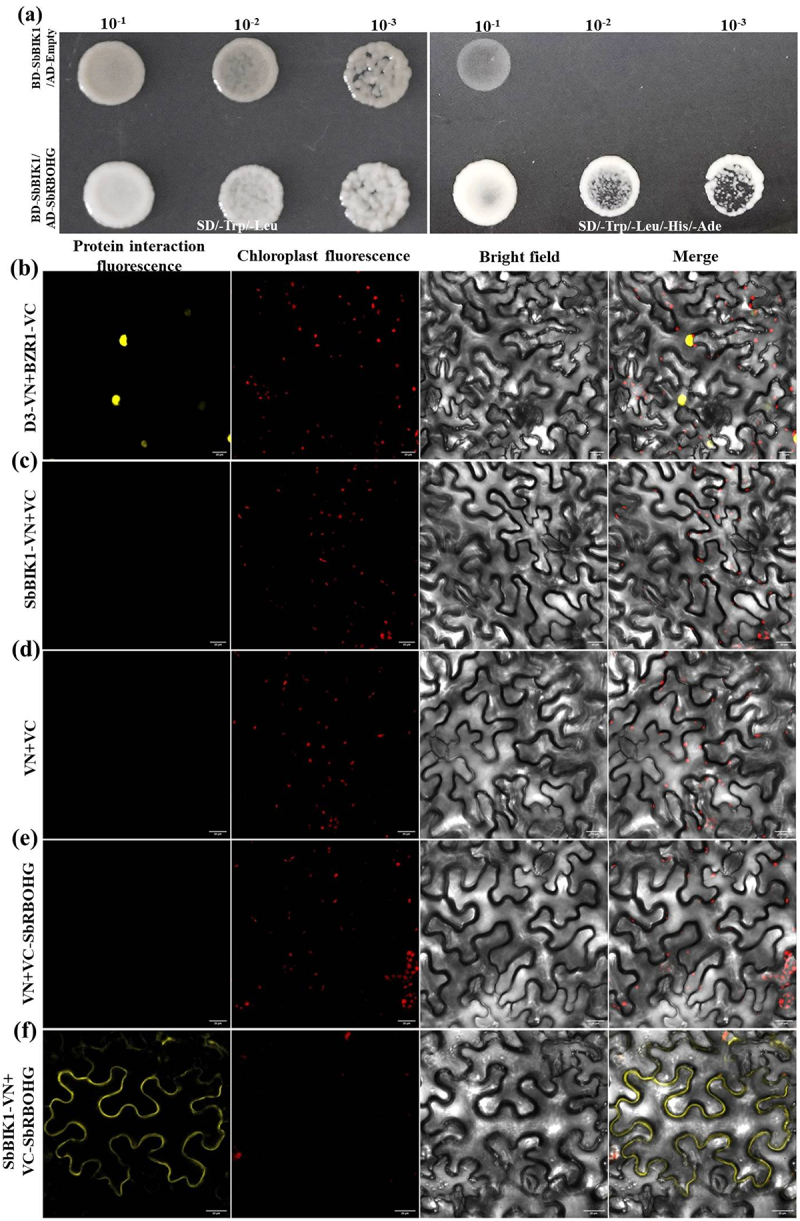
Validation of the interaction between sorghum SbBIK1 and SbRBOHG proteins. (a) Y2H analysis of SbBIK1 and SbRBOHG interaction. Top panel: SD/-Trp/-Leu medium (double-deficient medium) shows normal growth for all combination strains; Bottom panel: SD/-Trp/-Leu/-His/-Ade medium (quadruple-deficient medium) indicates only the BD-SbBIK1/AD-SbRBOHG combination exhibits growth. Lower panel: SD/-Trp/-Leu/-His/-Ade medium (quadruple-deficient medium) shows only the BD-SbBIK1/AD-SbRBOHG combination forming white colonies at dilutions ranging from 10^− 1^ to 10^− 3^, indicating a specific interaction between SbBIK1 and SbRBOHG. The empty vector control (BD-SbBIK1/AD-Empty) showed no growth; (b-f) BiFC validated the interaction between SbBIK1 and SbRBOHG in plant cells. From left to right: fluorescent protein signals (yellow), chloroplast auto-fluorescence (red), brightfield image, and multi-channel overlay image. (b) Positive control (D3-VN+BZR1-VC) shows nuclear fluorescence signal; (c) SbBIK1-VN+VC empty vector control exhibits no fluorescence signal; (d) SbBIK1-VN+SbRBOHG-VC combination displays distinct fluorescence signal in the plasma membrane region; (e) VN empty vector + SbRBOHG-VC control showed no fluorescence; (f) VN empty vector + VC empty vector control showed no fluorescence. Results indicate that SbBIK1 and SbRBOHG undergo specific interaction on the plant plasma membrane. Scale bar = 20 μm.

In plant immune signaling pathways, the interaction between the receptor-like kinase BKI1 and RBOH family proteins typically participates in regulating reactive oxygen species bursts. To validate the interaction between SbBIK1 and SbRBOHG in sorghum, this study employed Y2H and BiFC assays. Y2H results demonstrated a specific interaction between SbBIK1 and SbRBOHG within yeast cells. On SD/-Trp/-Leu double-deficient medium, all transformation combinations grew normally, confirming successful vector co-transformation. However, on SD/-Trp/-Leu/-His/-Ade quadruple-deficient medium, only the BD-SbBIK1/AD-SbRBOHG co-transformed combination formed white colonies at dilutions ranging from 10^−1^ to 10^−3^ (Figure 7(a)). The empty vector control (BD-SbBIK1/AD empty vector) showed no growth on the quadruple-deficient medium, ruling out the possibility of self-activation. These results confirm that SbBIK1 and SbRBOHG can directly bind, with interaction strength exhibiting concentration-dependent characteristics. This finding suggests SbBIK1 may regulate SbRBOHG’s enzymatic activity through direct interaction, providing molecular evidence for elucidating early signal transduction mechanisms in sorghum’s Al^3+^ stress response.

Fluorescence complementation experiments further validated the interaction characteristics and subcellular localization of SbBIK1 and SbRBOHG in vivo. In the SbBIK1-nYFPC and SbRBOHG-cYFPN co-expression system, strong yellow fluorescence signals were detected in the plasma membrane region of *N. benthamiana* epidermal cells (Figure 7(b-f)). This signal did not overlap with the chloroplast auto-fluorescence region, indicating that the interaction occurs at the plasma membrane. The positive control D3-VN+BZR1-VC showed nuclear fluorescence, while all negative control combinations exhibited no specific fluorescence (Figure 7(b)). Notably, the fluorescence signal generated by SbBIK1-SbRBOHG interaction displayed typical membrane-associated characteristics, forming a continuous distribution pattern along the cell periphery, consistent with the expected localization of both as membrane-associated proteins (Figure 7(f)). These findings not only validate the yeast two-hybrid results but also reveal the specific cellular compartment where the interaction occurs. This provides crucial cell biological evidence for elucidating the molecular mechanism by which the SbBIK1-SbRBOHG complex regulates Al^3+^ stress signaling through plasma membrane localization.

## 4. Discussion

**Figure 8.**
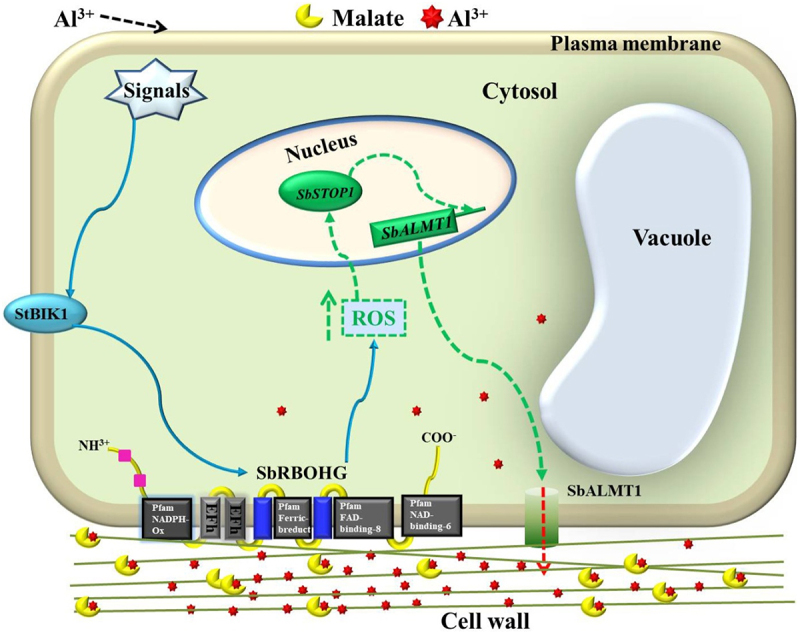
A molecular mechanism models of sorghum root cells responding to aluminum stress. When external Al^3+^ enters the cell wall region, an initial signal is triggered at the plasma membrane. This signal activates the serine/threonine protein kinase SbBIK1, which in turn promotes interaction between SbBIK1 and the plasma membrane-localized homolog of the respiratory burst oxidase SbRBOHG. Activated *SbRBOHG* exhibits enhanced NADPH oxidase activity, catalyzing substantial ROS production to amplify the stress signal. ROS further regulates the expression of the nuclear Al^3+^ stress response transcription factor *SbSTOP1*. Up-regulated *SbSTOP1* activates transcription of its downstream gene *SbALMT1*. The SbALMT1 protein forms an anion channel in the plasma membrane, mediating malate secretion into the extracellular space. The exported malate forms chelates with Al^3+^ in the cell wall region, generating stable, nontoxic complexes that alleviate Al^3+^ toxicity and enhance plant Al^3+^ tolerance.

Through a comprehensive genome-wide analysis, this study identified the *RBOH* gene family in sorghum, comprising 10 members designated *SbRBOHA* to *SbRBOHJ*. Chromosomal mapping revealed an uneven distribution pattern, with *SbRBOHC*, *SbRBOHD*, *SbRBOHE*, and *SbRBOHF* forming a distinct cluster on chromosome 3 (Figure S1) 54. Expression profiling demonstrated that *SbRBOHG* displays pronounced root-specific expression, suggesting its potential involvement in root-mediated stress adaptation (Figure 1(a)). Sequence alignment and structural modeling indicated that SbRBOHG shares 64.79% amino acid identity with *Arabidopsis* AtRBOHD and maintains a highly conserved three-dimensional conformation (Figure 2 and Figure S4). Functional analyses showed that heterologous overexpression of *SbRBOHG* in *Arabidopsis* significantly improved Al^3+^ stress tolerance, correlated with enhanced ROS accumulation in root apices (Figure 4). Protein interaction studies further established a direct physical interaction between SbRBOHG and the serine/threonine kinase SbBIK1 (Figure 7). Additionally, *SbRBOHG* overexpression up-regulated the expression of *SbSTOP1*—a central Al^3+^ stress regulator – and its downstream effector, the anion channel gene *SbALMT1* (Figure 6).These results delineate a proposed “SbBIK1→SbRBOHG→ROS→SbSTOP1→SbALMT1” signaling axis that underlies sorghum Al^3+^ tolerance (Figure 8), providing mechanistic insights into the molecular basis of crop adaptation to Al^3+^ toxicity.

Previous studies have demonstrated that *Arabidopsis AtRBOHD* and *AtRBOHF* primarily participate in biotic stress responses, whereas rice *OsRBOHF* plays a crucial role in abiotic stress adaptation.^20,47–49^ Consistent with these observations, *RBOH* genes from various plant species exhibit tissue-specific expression patterns. For example, tomato *SlRBOHB* shows high root-specific expression, and its overexpression enhances salt tolerance.^50^ However, unlike the previously described mechanism in *Arabidopsis* where ROS mediate STOP1 degradation through RAE1 regulation,^32^ the current study found that *SbRBOHG*-induced ROS accumulation coincided with up-regulated *SbSTOP1* expression, suggesting potential divergence in ROS signaling mechanisms among plant species (Figure 6). Notably, this study provides the first confirmation of a direct interaction between *SbBIK1* and *SbRBOHG* in sorghum (Figure 7), echoing the established BIK1-RBOHD module that regulates immune signaling in *Arabidopsis*. This conservation suggests that the interaction may be evolutionarily maintained between monocots and dicots. Nevertheless, distinct functional specializations are evident: while the *Arabidopsis* BIK1-RBOHD module primarily mediates pathogen-associated molecular pattern (PAMP)-triggered immunity ^46^ Yuan et al. 2024, the SbBIK1-SbRBOHG module appears specifically involved in Al^3+^ stress signaling (Figure 7 and 8). These comparative analyses not only validate the central role of RBOH proteins in plant stress responses but also highlight the existence of species-specific regulatory networks. The coexistence of functional conservation and species specificity likely reflects evolutionary adaptations to diverse environmental pressures. While the core biochemical function of RBOH proteins has been maintained throughout evolution, their regulatory circuits have undergone lineage-specific modifications to meet particular ecological demands. These insights advance our understanding of plant stress response evolution and provide valuable guidance for selecting target genes in breeding stress-tolerant crops. The conservation of key signaling components across species suggests potential for knowledge transfer between model systems and crops, while the observed specificity underscores the importance of validating mechanisms in target species. The molecular characterization of sorghum’s Al stress response pathway provides a framework for understanding how monocot crops perceive and respond to soil toxicity. Further research should explore whether similar mechanisms operate in other economically important cereals, potentially enabling the development of broad-spectrum stress tolerance in staple crops.

In *Arabidopsis*, the plasma membrane-localized RBOHD, as a member of the NADPH oxidase family, serves as a key source of ROS bursts in plants and plays a central role in plant immunity and abiotic stress responses,^51–53^ In recent years, its function in Al^3+^ stress responses has gradually attracted attention, yet its specific mechanism remains highly controversial.^32,36,37^ One study proposed that the Al^3+^ sensor ALR1/PSKR1 mediates RBOHD phosphorylation and activation via the co-receptor BAK1, thereby inducing ROS production.^32^ The generated ROS oxidatively modifies RAE1, a degradation regulator of the STOP1 protein, stabilizing STOP1 levels and activating downstream Al^3+^ tolerance related gene expression, ultimately enhancing plant Al^3+^ tolerance.^32^ This model is supported by genetic evidence, as *rbohD* mutants exhibit increased Al^3+^ sensitivity and reduced STOP1 accumulation. However, a recent study challenges this model, proposing that H_2_O_2_ derived from RBOHD negatively regulates STOP1 accumulation and Al^3+^ tolerance.^37^ Corresponding experiments show that *rbohD* mutants instead exhibit a slight increase in Al^3+^ tolerance and higher STOP1 accumulation. Further genetic analysis indicates that overexpressing catalase (e.g., *CAT2*) to reduce H_2_O_2_ levels enhances Al^3+^ tolerance in a RAE1-dependent manner.^37^ These contradictory findings suggest that the RBOHD-ROS signaling module may exhibit context-dependent regulatory functions in Al^3+^ stress responses, with its specific mechanisms awaiting systematic elucidation.

Notably, functional studies on sorghum SbRBOHG (homologous to AtRBOHD) revealed that its overexpression, while enhancing ROS bursts under Al^3+^ stress, unexpectedly reduced root tip Al^3+^ accumulation contradicting the conventional view that ROS exacerbates metal toxicity (Fig. 4). Further studies have shown that under aluminum stress conditions, Arabidopsis plants heterologously expressing SbRBOHG not only exhibited increased NADPH oxidase activity and H_2_O_2_ accumulation, but also demonstrated up-regulation of the key aluminum tolerance genes AtSTOP1 and AtALMT1. Furthermore, this study confirmed that SbRBOHG physically interacts with the kinase SbBIK1 on the plasma membrane; however, the specific signaling mediators and transduction pathways involved remain to be elucidated. Therefore, future work should further elucidate the precise functions of SbRBOHG in specific cellular compartments and aluminum signaling pathways, particularly by systematically elucidating how the ROS they generate interact with regulatory modules such as STOP1-RAE1 to achieve dynamic and fine-tuned regulation within the aluminum stress response network.

From the above, ROS plays far more than a single role in plant Al^3+^ stress responses, exhibiting significant context dependence and pathway specificity. Elucidating its specific regulatory mechanisms across different species, tissues, and signaling pathways holds substantial theoretical value for genetic improvement of crop Al^3+^ tolerance. Our findings significantly expand the theoretical framework of Al^3+^ stress signaling pathways. The classical “ROS burst→transcriptional reprogramming” model posits that Al^3+^ trigger increased activity of the plasma membrane RBOH, which then modulates transcription factor stability through oxidative modifications. The proposed “SbBIK1→SbRBOHG→ ROS→SbSTOP1→SbALMT1” cascade pathway aligns closely with this model while introducing a kinase-mediated precision control layer: SbBIK1 likely activates SbRBOHG through phosphorylation, enhancing the spatiotemporal specificity of ROS production (Figure 8). This finding strongly complements the BIK1→RBOHD module model regulating immune signaling in *Arabidopsis*, suggesting that kinase-oxidase coupling may represent a conserved mechanism in plant stress signaling.^37^ Furthermore, the synchronized up-regulation of *SbSTOP1* with ROS accumulation supports the hypothesis that ROS acts as a second messenger to directly or indirectly regulate transcriptional activity. However, unlike previous studies by our group, the activation level of *SbALMT1* does not show a perfect linear correlation with ROS levels, suggesting potential feedback regulation or branching pathways. These findings necessitate revisions to existing theoretical models, emphasizing the networked nature of signaling pathways rather than simple linear relationships.

There are several limitations in this study that warrant attention. First, functional validation primarily relied on the *Arabidopsis* heterologous overexpression system, lacking verification in the native genetic background of sorghum. This may affect the accuracy of interpreting the physiological function of *SbRBOHG*. The heterologous expression system may lack sorghum specific interacting proteins or regulatory factors, potentially influencing phenotypic interpretation. Second, although direct interaction between SbBIK1 and SbRBOHG was confirmed, the specific interaction domains, key phosphorylation sites, and their biological functions remain unresolved, hindering a deeper understanding of the regulatory mechanism. Third, the study primarily focused on Al^3+^ stress responses, leaving unresolved whether SbRBOHG participates in other abiotic stresses (e.g., drought, salt stress), and its functional universality requires further validation. Additionally, the relatively high Al^3+^ treatment concentrations used in experiments may not fully mimic complex field soil environments, limiting the practical applicability of the findings. Finally, the association analysis between omics data and phenotypes largely relies on correlation-based inference, lacking direct genetic evidence (e.g., phenotypic validation of gene-edited mutants). Future work should integrate multi-omics data and employ precise genetic manipulation to further validate causal relationships.

Based on the current findings and limitations, future research should prioritize the following directions: First, establish CRISPR/Cas9-edited sorghum *SbRBOHG* materials to validate its function in its native genetic background, while systematically analyzing its regulatory network through transcriptomics and metabolomics. Second, elucidate the molecular mechanism of phosphorylation regulation by determining the three dimensional structure of the SbBIK1-SbRBOHG interaction interface using structural biology methods. Additionally, SbRBOHG cross talk mechanisms in integrating multiple stress signals (e.g., aluminum toxicity, drought, low temperature) should be explored to reveal its functional plasticity under complex environmental conditions. Concurrently, key genes identified in this study (e.g., *SbSTOP1*, *SbALMT*1) can be applied to molecular design breeding. Through polygenic aggregation strategies, new crop varieties with broad spectrum stress tolerance can be developed. These studies will not only advance the theoretical framework of plant stress biology but also provide crucial technological support for sustainable agricultural development.

## 5. Conclusion

In summary, this study identified 10 RBOH family members in the sorghum genome, distributed across seven chromosomes and exhibiting species-specific evolutionary characteristics. Among them, *SbRBOHG* showed significantly high expression in root tissues, suggesting its potential involvement in root stress responses. Although SbRBOHG shares only 64.79% sequence identity with *Arabidopsis* AtRBOHD, both proteins exhibit high conservation in their three-dimensional structure and functional domain composition, suggesting functional universality. Functional experiments demonstrate that heterologous overexpression of *SbRBOHG* enhances *Arabidopsis* tolerance to Al^3+^ stress, manifested as alleviated root growth inhibition, increased biomass, and reduced root tip Al accumulation. This phenotype may be related to a ROS-mediated aluminum chelation mechanism. Early Al^3+^ stress induces increased NADPH oxidase activity and H_2_O_2_ accumulation, while heterologous overexpression of *SbRBOHG* further amplifies this ROS burst, suggesting that it enhances the plant’s response to Al^3+^ toxicity through a positive feedback mechanism. At the molecular level, SbRBOHG directly interacts physically with the kinase SbBIK1 on the plasma membrane, as verified by Y2H and BiFC assays. Additionally, heterologous overexpression of *SbRBOHG* up-regulates the expression of the *Arabidopsis* Al^3+^ stress-related transcription factor *AtSTOP1* and its downstream transporter gene *AtALMT1*. This study provides preliminary insights into the role of SbRBOHG in Al^3+^ toxicity and offers novel perspectives on the ROS regulatory network response to Al stress in non-model crops.

## Supplementary Material

Supporting Information .docx

## References

[1] Mittler R, Zandalinas SI, Fichman Y, Van Breusegem F. Reactive oxygen species signalling in plant stress responses. Nat Rev Mol Cell Biol. 2022;23(10):663–24. doi: 10.1038/s41580-022-00499-2. 35760900

[2] Zhang X, Zhang D, Zhong C, Li W, Dinesh-Kumar SP, Zhang Y. Orchestrating ROS regulation: Coordinated post-translational modification switches in NADPH oxidases. The New Phytol. 2025;245(2):510–22. doi: 10.1111/nph.20231. 39468860

[3] Hafsi C, Collado-Arenal AM, Wang H, Sanz-Fernández M, Sahrawy M, Shabala S, Romero-Puertas MC, Sandalio LM. The role of NADPH oxidases in regulating leaf gas exchange and ion homeostasis in Arabidopsis plants under cadmium stress. J Retailing Hazard Mater. 2022;429:128217. doi: 10.1016/j.jhazmat.2022.128217. 35077969

[4] Mittal M, Siddiqui MR, Tran K, Reddy SP, Malik AB. Reactive oxygen species in inflammation and tissue injury. Antioxid Redox Signal. 2014;20(7):1126–67. doi: 10.1089/ars.2012.5149. 23991888 PMC3929010

[5] Raad H, Paclet MH, Boussetta T, Kroviarski Y, Morel F, Quinn MT, Gougerot-Pocidalo M-A, Dang PMC, El-Benna J. Regulation of the phagocyte NADPH oxidase activity: phosphorylation of gp91phox/NOX2 by protein kinase C enhances its diaphorase activity and binding to Rac2, p67phox, and p47phox. Faseb J. 2009;23(4):1011–22. doi: 10.1096/fj.08-114553. 19028840 PMC2660639

[6] Sagi M, Davydov O, Orazova S, Yesbergenova Z, Ophir R, Stratmann JW, Fluhr R. Plant respiratory burst oxidase homologs impinge on wound responsiveness and development in Lycopersicon esculentum [w]. Plant Cell. 2004;16(3):616–28. doi: 10.1105/tpc.019398. 14973161 PMC385276

[7] Marino D, Andrio E, Danchin EG, Oger E, Gucciardo S, Lambert A, Puppo A, Pauly N. A Medicago truncatula NADPH oxidase is involved in symbiotic nodule functioning. The New Phytol. 2011;189(2):580–92. doi: 10.1111/j.1469-8137.2010.03509.x. 21155825 PMC3491693

[8] Kobayashi M, Kawakita K, Maeshima M, Doke N, Yoshioka H. Subcellular localization of strboh proteins and NADPH-dependent O2(-)-generating activity in potato tuber tissues. J Exp Botany. 2006;57(6):1373–79. doi: 10.1093/jxb/erj113. 16551687

[9] Suzuki N, Mittler R. Reactive oxygen species-dependent wound responses in animals and plants. Free Radical Biol Med. 2012;53(12):2269–76. doi: 10.1016/j.freeradbiomed.2012.10.538. 23085520

[10] Kobayashi M, Ohura I, Kawakita K, Yokota N, Fujiwara M, Shimamoto K, Doke N, Yoshioka H. Calcium-dependent protein kinases regulate the production of reactive oxygen species by potato NADPH oxidase. Plant Cell. 2007;19(3):1065–80. doi: 10.1105/tpc.106.048884. 17400895 PMC1867354

[11] Groom QJ, Torres MA, Fordham-Skelton AP, Hammond-Kosack KE, Robinson NJ, Jones JDG. RbohA, a rice homologue of the mammalian gp91phox respiratory burst oxidase gene. The Plant J: Cell Mol Biol. 1996;10(3):515–22. doi: 10.1046/j.1365-313x.1996.10030515.x. 8811865

[12] Shim E, Lee JW, Park H, Zuccarello GC, Kim GH. Hydrogen peroxide signalling mediates fertilization and post-fertilization development in the red alga Bostrychia moritziana. J Exp Botany. 2022;73(3):727–41. doi: 10.1093/jxb/erab453. 34652437

[13] Zhang H, Wang X, Yan A, Deng J, Xie Y, Liu S, Liu D, He L, Weng J, Xu J. Evolutionary analysis of respiratory burst oxidase homolog (RBOH) genes in plants and characterization of ZmRBOHs. Int J Mol Sci. 2023;24(4):3858. doi: 10.3390/ijms24043858. 36835269 PMC9965149

[14] Sagi M, Fluhr R. Production of reactive oxygen species by plant NADPH oxidases. Plant Physiol. 2006;141(2):336–40. doi: 10.1104/pp.106.078089. 16760484 PMC1475462

[15] Wang GF, Li WQ, Li WY, Wu G-L, Zhou C-Y, Chen K-M. Characterization of rice NADPH oxidase genes and their expression under various environmental conditions. Int J Mol Sci. 2013;14(5):9440–58. doi: 10.3390/ijms14059440. 23629674 PMC3676792

[16] Yu S, Kakar KU, Yang Z, Nawaz Z, Lin S, Guo Y, Ren X-L, Baloch AA, Han D. Systematic study of the stress-responsive Rboh gene family in Nicotiana tabacum: genome-wide identification, evolution and role in disease resistance. Genomics. 2020;112(2):1404–18. doi: 10.1016/j.ygeno.2019.08.010. 31430516

[17] Liu J, Lu H, Wan Q, Qi W, Shao H. Genome-wide analysis and expression profiling of respiratory burst oxidase homologue gene family in Glycine max. Environ Exp Botany. 2019;161 13. doi: 10.1016/j.envexpbot.2018.07.015.

[18] Hu CH, Wei XY, Yuan B, Yao L-B, Ma T-T, Zhang P-P, Wang X, Wang P-Q, Liu W-T, Li W-Q, et al. Genome-wide identification and functional analysis of NADPH oxidase family genes in wheat during development and environmental stress responses. Front Plant Sci. 2018;9:906. doi: 10.3389/fpls.2018.00906. 30083172 PMC6065054

[19] Kong L, Ma X, Zhang C, Kim S-I, Li B, Xie Y, Yeo I-C, Thapa H, Chen S, Devarenne TP, et al. Dual phosphorylation of DGK5-mediated PA burst regulates ROS in plant immunity. Cell. 2024;187(3):609–23.e21. doi: 10.1016/j.cell.2023.12.030. 38244548 PMC10872252

[20] Liu Y, He C. Regulation of plant reactive oxygen species (ROS) in stress responses: learning from AtRBOHD. Plant Cell Rep. 2016;35(5):995–1007. doi: 10.1007/s00299-016-1950-x. 26883222

[21] Luo X, Dai Y, Zheng C, Yang Y, Chen W, Wang Q, Chandrasekaran U, Du J, Liu W, Shu K. The abi4-RbohD/VTC2 regulatory module promotes reactive oxygen species (ROS) accumulation to decrease seed germination under salinity stress. The New Phytol. 2021;229(2):950–62. doi: 10.1111/nph.16921. 32916762

[22] Horemans N, Raeymaekers T, Van Beek K, Nowocin A, Blust R, Broos K, Cuypers A, Vangronsveld J, Guisez Y. Dehydroascorbate uptake is impaired in the early response of Arabidopsis plant cell cultures to cadmium. J Exp Botany. 2007;58(15–16):4307–17. doi: 10.1093/jxb/erm291. 18182433

[23] Jiang C, Belfield EJ, Cao Y, Smith JAC, Harberd NP. An Arabidopsis soil-salinity-tolerance mutation confers ethylene-mediated enhancement of sodium/potassium homeostasis. Plant Cell. 2013;25(9):3535–52. doi: 10.1105/tpc.113.115659. 24064768 PMC3809548

[24] Toum L, Torres PS, Gallego SM, Benavídes MP, Vojnov AA, Gudesblat GE. Coronatine inhibits stomatal closure through Guard Cell-specific inhibition of NADPH oxidase-dependent ROS production. Front Plant Sci. 2016;7:1851. doi: 10.3389/fpls.2016.01851. 28018388 PMC5155495

[25] Müller K, Carstens AC, Linkies A, Torres MA, Leubner-Metzger G. The NADPH-oxidase AtrbohB plays a role in Arabidopsis seed after-ripening. The New Phytol. 2009;184(4):885–97. doi: 10.1111/j.1469-8137.2009.03005.x. 19761445

[26] Carol RJ, Takeda S, Linstead P, Durrant MC, Kakesova H, Derbyshire P, Drea S, Zarsky V, Dolan L. A RhoGDP dissociation inhibitor spatially regulates growth in root hair cells. Nature. 2005;438(7070):1013–16. doi: 10.1038/nature04198. 16355224

[27] Kuběnová L, Tichá M, Šamaj J, Ovečka M. Root hair defective 2 vesicular delivery to the apical plasma membrane domain during Arabidopsis root hair development. Plant Physiol. 2022;188(3):1563–85. doi: 10.1093/plphys/kiab595. 34986267 PMC8896599

[28] Dai C, Lee Y, Lee IC, Nam HG, Kwak JM. Calmodulin 1 regulates senescence and ABA response in Arabidopsis. Front Plant Sci. 2018;9:803. doi: 10.3389/fpls.2018.00803. 30013580 PMC6036150

[29] Kaya H, Takeda S, Kobayashi MJ, Kimura S, Iizuka A, Imai A, Hishinuma H, Kawarazaki T, Mori K, Yamamoto Y, et al. Comparative analysis of the reactive oxygen species-producing enzymatic activity of Arabidopsis NADPH oxidases. The Plant J: Cell Mol Biol. 2019;98(2):291–300. doi: 10.1111/tpj.14212. 30570803

[30] Zhang W, Munyaneza V, Yi B, Zhang X, Zhang P, Kant S, Xu F, Ding G. Tolerance to aluminum toxicity in Brassica napus is mediated by regulating cell wall Al sequestration, antioxidant defense, and metabolic adaptation. J Retailing Hazard Mater. 2025;498:139837. Advance online publication. 10.1016/j.jhazmat.2025.139837. 40961705

[31] Liu H, Bai C, Cai J, Wu Y, Zhu C. Yucasin alleviates aluminum toxicity associated with regulating reactive oxygen species homeostasis in tomato seedlings. Toxics. 2025;13(5):406. doi: 10.3390/toxics13050406. 40423485 PMC12115678

[32] Ding ZJ, Xu C, Yan JY, Wang YX, Cui MQ, Yuan JJ, Wang YN, Li GX, Wu JX, Wu YR, et al. The LRR receptor-like kinase ALR1 is a plant aluminum ion sensor. Cell Res. 2024;34(4):281–94. doi: 10.1038/s41422-023-00915-y. 38200278 PMC10978910

[33] Fu XZ, Wang X, Liu JJ, Chen Y-X, Wang A-Q, Zhan J, Han Z-Q, He L-F, Xiao D. AhASRK1, a peanut dual-specificity kinase that activates the Ca^2+^-ROS-MAPK signalling cascade to mediate programmed cell death induced by aluminium toxicity via ABA. Plant Physiol Biochem: PPB. 2025;220:109538. doi: 10.1016/j.plaphy.2025.109538. 39864296

[34] Matsumoto H, Motoda H. Aluminum toxicity recovery processes in root apices. Possible association with oxidative stress. Plant Sci. 2012;185-186:1–8. doi: 10.1016/j.plantsci.2011.07.019. 22325861

[35] Wang S, Cheng H, Wei Y. Supplemental silicon and boron alleviates aluminum-induced oxidative damage in soybean roots. Plants (Basel, Switz). 2024;13(6):821. doi: 10.3390/plants13060821. PMC10975118 38592832

[36] Huang CF, Ma Y. Aluminum resistance in plants: a critical review focusing on STOP1. Plant Commun. 2025;6(2):101200. doi: 10.1016/j.xplc.2024.101200. 39628052 PMC11897453

[37] Wei X, Zhu Y, Xie W, Ren W, Zhang Y, Zhang H, Dai S, Huang C-F. H2O2 negatively regulates aluminum resistance via oxidation and degradation of the transcription factor STOP1. Plant Cell. 2024;36(3):688–708. doi: 10.1093/plcell/koad281. 37936326 PMC10896299

[38] Huang L, Li H, Luo Y, Shi J, Kong L, Teng W. Exogenous silicon alleviates aluminum stress in eucalyptus species by enhancing the antioxidant capacity and improving plant growth and tolerance quality. BMC Plant Biol. 2024;24(1):997. doi: 10.1186/s12870-024-05723-z. 39443879 PMC11515708

[39] Zhang F, Yan X, Han X, Tang R, Chu M, Yang Y, Yang Y-H, Zhao F, Fu A, Luan S, et al. A defective vacuolar proton pump enhances aluminum tolerance by reducing vacuole sequestration of organic acids. Plant Physiol. 2019;181(2):743–61. doi: 10.1104/pp.19.00626. 31350362 PMC6776860

[40] Jeppe H, Tsirigos KD, Pedersen MD, Almagro Armenteros JJ, Marcatili P, Nielsen H, Krogh A, Winther O. DeepTMHMM predicts alpha and beta transmembrane proteins using deep neural networks. 2022; doi: 10.1101/2022.04.08.487609.

[41] Livak KJ, Schmittgen TD. Analysis of relative gene expression data using real-time quantitative PCR and the 2(-delta delta C(t)) method. Methods (San Diego, Calif). 2001;25(4):402–08. doi: 10.1006/meth.2001.1262. 11846609

[42] Fleming J, Magana P, Nair S, Tsenkov M, Bertoni D, Pidruchna I, Lima Afonso MQ, Midlik A, Paramval U, Žídek A, et al. Alphafold protein structure database and 3d-beacons: new data and capabilities. J Educ Chang Mol Biol. 2025;437(15):168967. doi: 10.1016/j.jmb.2025.168967. 40133787

[43] Chen J, Li H, Yang K, Wang Y, Yang L, Hu L, Liu R, Shi Z. Melatonin facilitates lateral root development by coordinating PAO-derived hydrogen peroxide and Rboh-derived superoxide radical. Free Radical Biol Med. 2019;143:534–44. doi: 10.1016/j.freeradbiomed.2019.09.011. 31520769

[44] Du L, Jiang Z, Zhou Y, Shen L, He J, Xia X, Zhang L, Yang X. Genome-wide identification and expression analysis of respiratory burst oxidase homolog (RBOH) gene family in eggplant (Solanum melongena L.) under abiotic and biotic stress. Genes. 2023;14(9):1665. doi: 10.3390/genes14091665. 37761805 PMC10531080

[45] Bai J, Zhou Y, Sun J, Chen K, Han Y, Wang R, Zou Y, Du M, Lu D. Bik1 protein homeostasis is maintained by the interplay of different ubiquitin ligases in immune signaling. Nat Commun. 2023;14(1):4624. doi: 10.1038/s41467-023-40364-0. 37532719 PMC10397244

[46] Li L, Li M, Yu L, Zhou Z, Liang X, Liu Z, Cai G, Gao L, Zhang X, Wang Y, et al. The FLS2-associated kinase BIK1 directly phosphorylates the NADPH oxidase RbohD to control plant immunity. Cell Host Microbe. 2014;15(3):329–38. doi: 10.1016/j.chom.2014.02.009. 24629339

[47] Postiglione AE, Muday GK. The role of ROS homeostasis in ABA-induced guard cell signaling. Front Plant Sci. 2020;11:968. doi: 10.3389/fpls.2020.00968. 32695131 PMC7338657

[48] Swetha R, Sridhanya VM, Varanavasiappan S, Kumar KK, Kokiladevi E, Ravichandran V, Ramalingam J, Sudhakar D, Arul L. Root apoplastic barrier mechanism: an adaptive strategy to protect against salt stress. Mol Biol Rep. 2024;52(1):56. doi: 10.1007/s11033-024-10171-x. 39690255

[49] Tao H, Wang R, He F, Zhang C, Jiang S, Wang M, Xu X, Wang J, You X, Wang D, et al. Phosphorylation and ubiquitination synergistically promote the degradation of OsRbohB to modulate rice immunity. Plant Cell. 2025;37(12):koaf276. 10.1093/plcell/koaf276. 41248081

[50] Li X, Zhang H, Tian L, Huang L, Liu S, Li D, Song F. Tomato SlRbohB, a member of the NADPH oxidase family, is required for disease resistance against Botrytis cinerea and tolerance to drought stress. Front Plant Sci. 2015;6:463. doi: 10.3389/fpls.2015.00463. 26157450 PMC4477072

[51] Kadota Y, Sklenar J, Derbyshire P, Stransfeld L, Asai S, Ntoukakis V, Jones J, Shirasu K, Menke F, Jones A, et al. Direct regulation of the NADPH oxidase RBOHD by the PRR-associated kinase BIK1 during plant immunity. Mol Cell. 2014;54(1):43–55. doi: 10.1016/j.molcel.2014.02.021. 24630626

[52] Yuan M, Jiang Z, Bi G, Nomura K, Liu M, Wang Y, Cai B, Zhou J-M, He SY, Xin X-F. Pattern-recognition receptors are required for NLR-mediated plant immunity. Nature. 2021;592(7852):105–09. doi: 10.1038/s41586-021-03316-6. 33692546 PMC8016741

[53] Yu X, Liu Z, Qin A, Zhou Y, Zhao Z, Yang J, Hu M, Liu H, Liu Y, Sun S, et al. Fls2-rbohd module regulates changes in the metabolome of Arabidopsis in response to abiotic stress. Plant Environ Interact (Hoboken, NJ). 2023;4(1):36–54. doi: 10.1002/pei3.10101. PMC10168046 37284598

[54] Liu M, Zhang Y, Pan T, Li Y, Hong Y, Chen W, Yang Y, Zhao G, Shabala S, Yu M. Genome-wide analysis of respiratory burst oxidase homolog gene family in pea (Pisum sativum L.). Front Plant Sci. 2023;14:1321952. doi: 10.3389/fpls.2023.1321952. 38155848 PMC10754532

