# Supplementary material for: Analysis of the sorghum RBOH gene family and molecular mechanism of its member SbRBOHG in response to aluminum stress
Source: GM Crops Food. 2026 Apr 13;17(1):2655482. doi: 10.1080/21645698.2026.2655482 (PMC13078204; doi:10.1080/21645698.2026.2655482)
Supplement: Supporting Information .docx [file KGMC_A_2655482_SM6831.docx]

**The following Supporting Information is available for this article:**

**Figure S1** Chromosomal distribution of the sorghum *SbRBOH* gene family.

**Figure S2** Phylogenetic evolution, conserved domains, and gene structure of the sorghum *SbRBOH* gene family.

**Figure S3** Chromosomal distribution and colinearity analysis of the sorghum *SbRBOH* gene family.

**Figure S4** Amino acid sequence alignment analysis of sorghum SbRBOHG and its homologous proteins AtRBOHD and AtRBOHF in *Arabidopsis*.

**Figure S5** RT-PCR Characterization of heterologous SbRBOHG overexpressing sorghum lines.

**Table S1** Primers used in this study.

**Table S2** The naming and molecular characteristics of *SbRBOH* gene family members in sorghum.


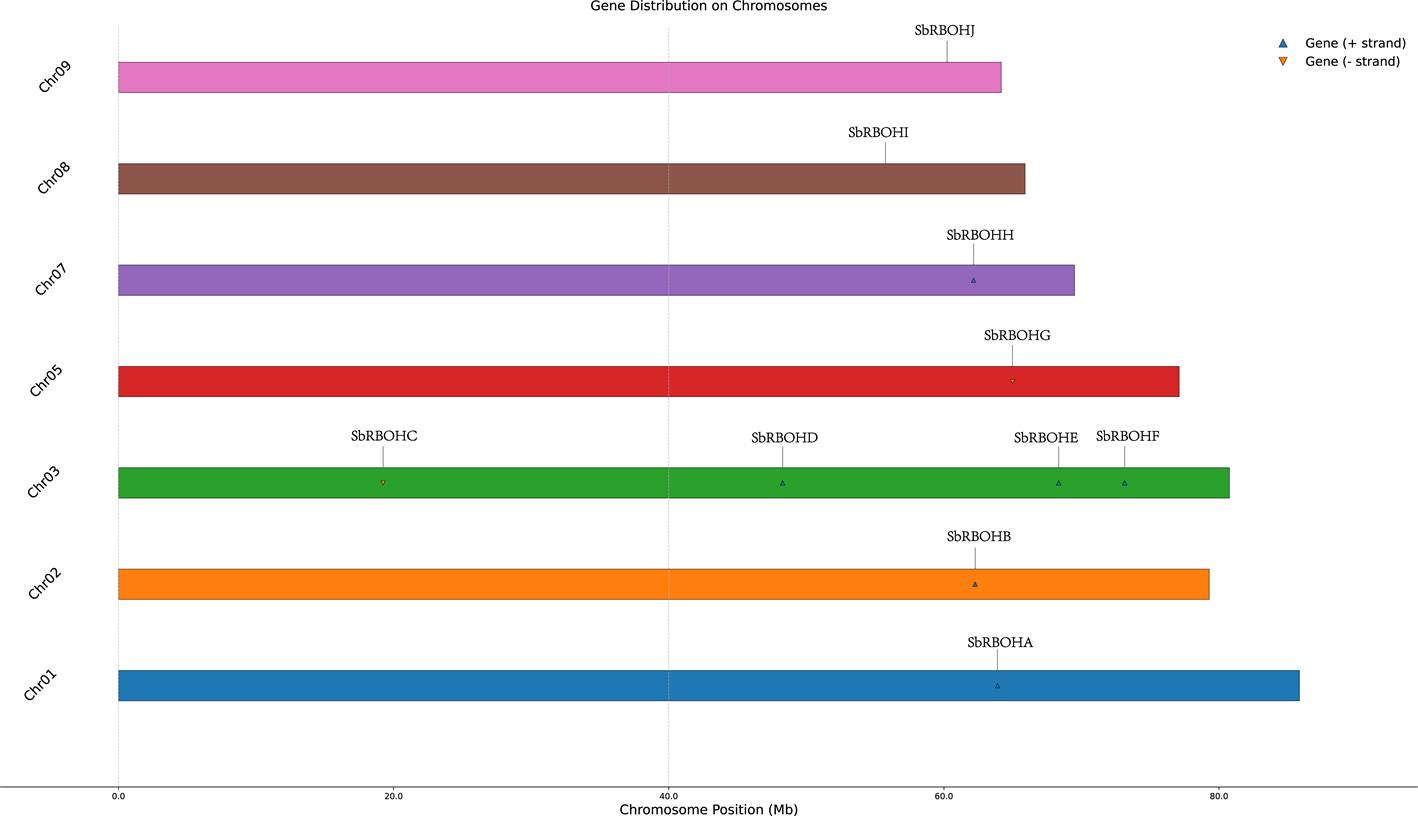


**Figure S1** Chromosomal distribution of the sorghum *SbRBOH* gene family. The 10 *SbRBOH* gene members identified in the sorghum genome are unevenly distributed across seven chromosomes. The horizontal axis represents chromosomal position (in Mb), while the vertical axis lists chromosome numbers (Chr01–Chr09). Each chromosome is depicted as a distinct colored band, with triangular markers indicating the physical locations of individual SbRBOH genes. Blue triangles denote genes located on the positive strand (+), while orange triangles indicate genes on the negative strand (-).


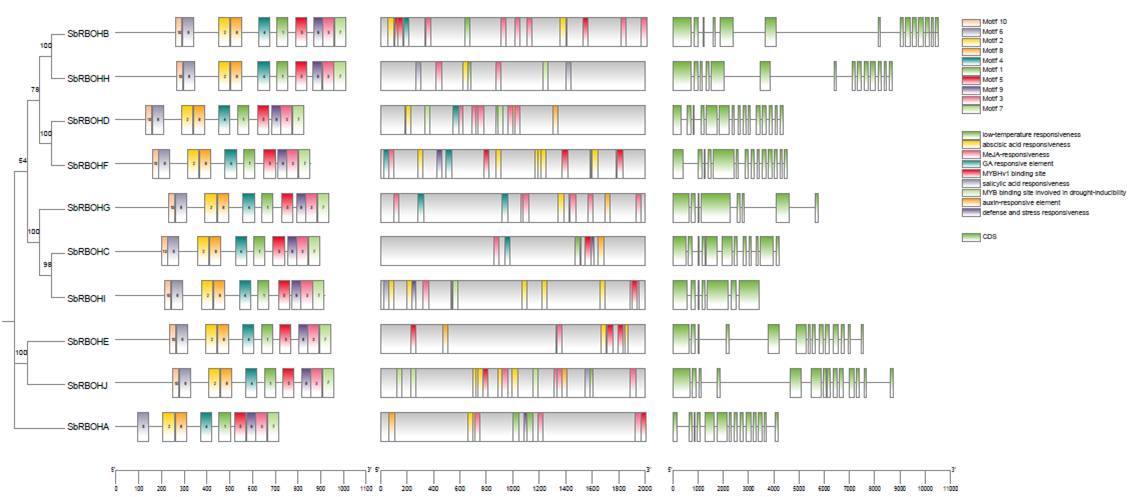


**Figure S2** Phylogenetic evolution, conserved domains, and gene structure of the sorghum *SbRBOH* gene family. (a) The phylogenetic tree constructed based on protein sequences shows that the 10 *SbRBOH* members can be divided into three major evolutionary branches, indicating that this family has undergone differentiation and expansion during evolution. (b) Analysis of conserved domains reveals that all members contain the typical NADPH oxidase core domain, including transmembrane domains, FAD-binding domains, and NADPH-binding domains. However, differences exist in the regulatory structures at the N- and C-termini within the cytoplasm (e.g., EF-hand, Ca^2+^-binding domains), suggesting potential functional diversification. (c) Schematic diagrams of gene structures reveal diversity among members in coding sequence (CDS) length, exon number, and intron distribution. Some members (e.g., *SbRBOHD* and *SbRBOHF*) exhibit similar intron-exon patterns consistent with their clustering relationships in the phylogenetic tree.


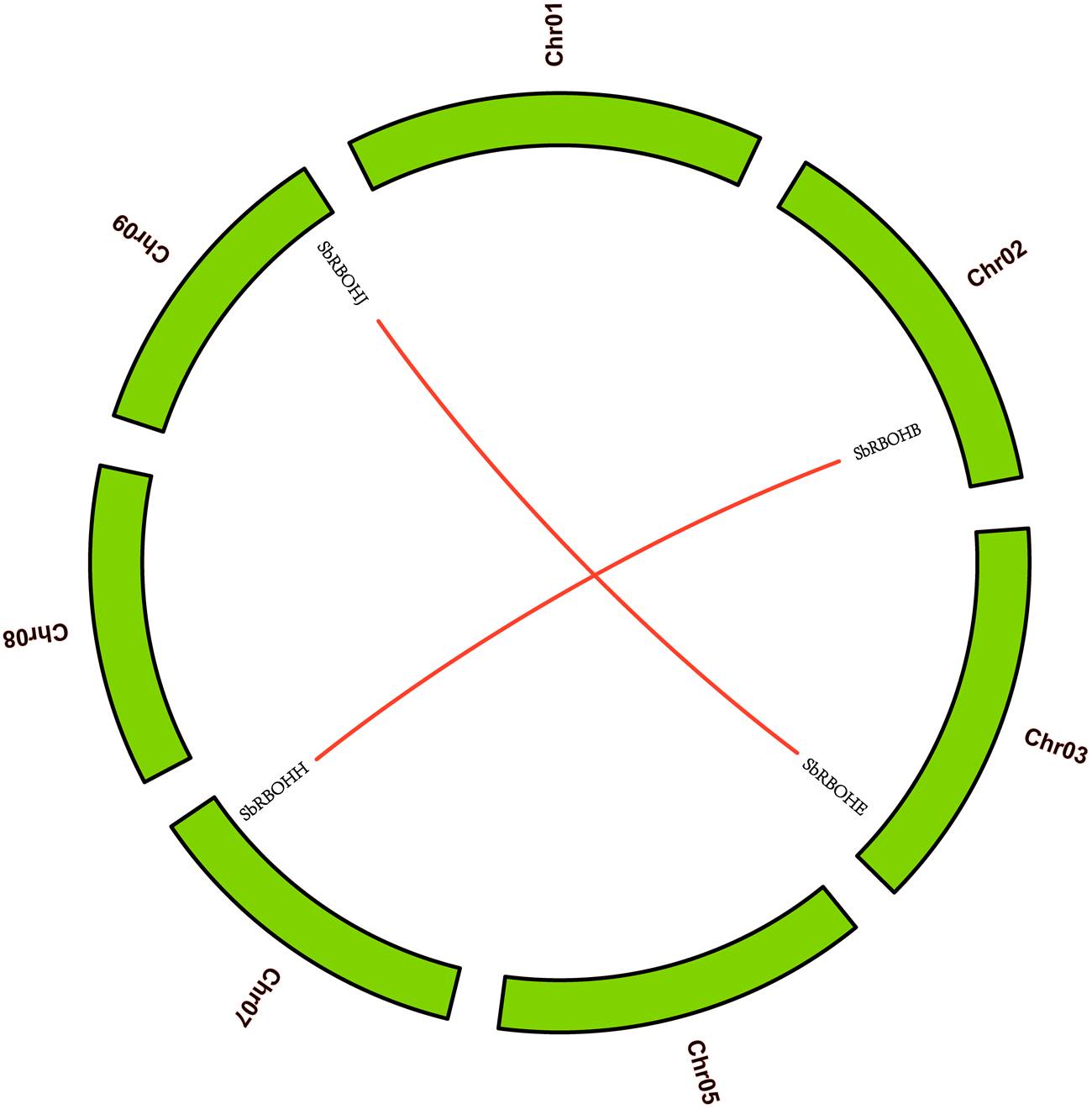


**Figure S3** Chromosomal distribution and colinearity analysis of the sorghum *SbRBOH* gene family. The circular layout displays the distribution of *SbRBOH* gene family members across sorghum's seven chromosomes. Chromosomes (Chr01–Chr09) are represented by green bands arranged uniformly around the circumference; Red lines indicate the specific chromosomal positions of individual SbRBOH genes (e.g., *SbRBOHA*, *SbRBOHB*) and suggest potential evolutionary or regulatory associations. The distribution pattern reveals uneven distribution of SbRBOH members across chromosomes, with multiple genes present on some chromosomes, implying that the family may have expanded through segmental duplication or local amplification events.


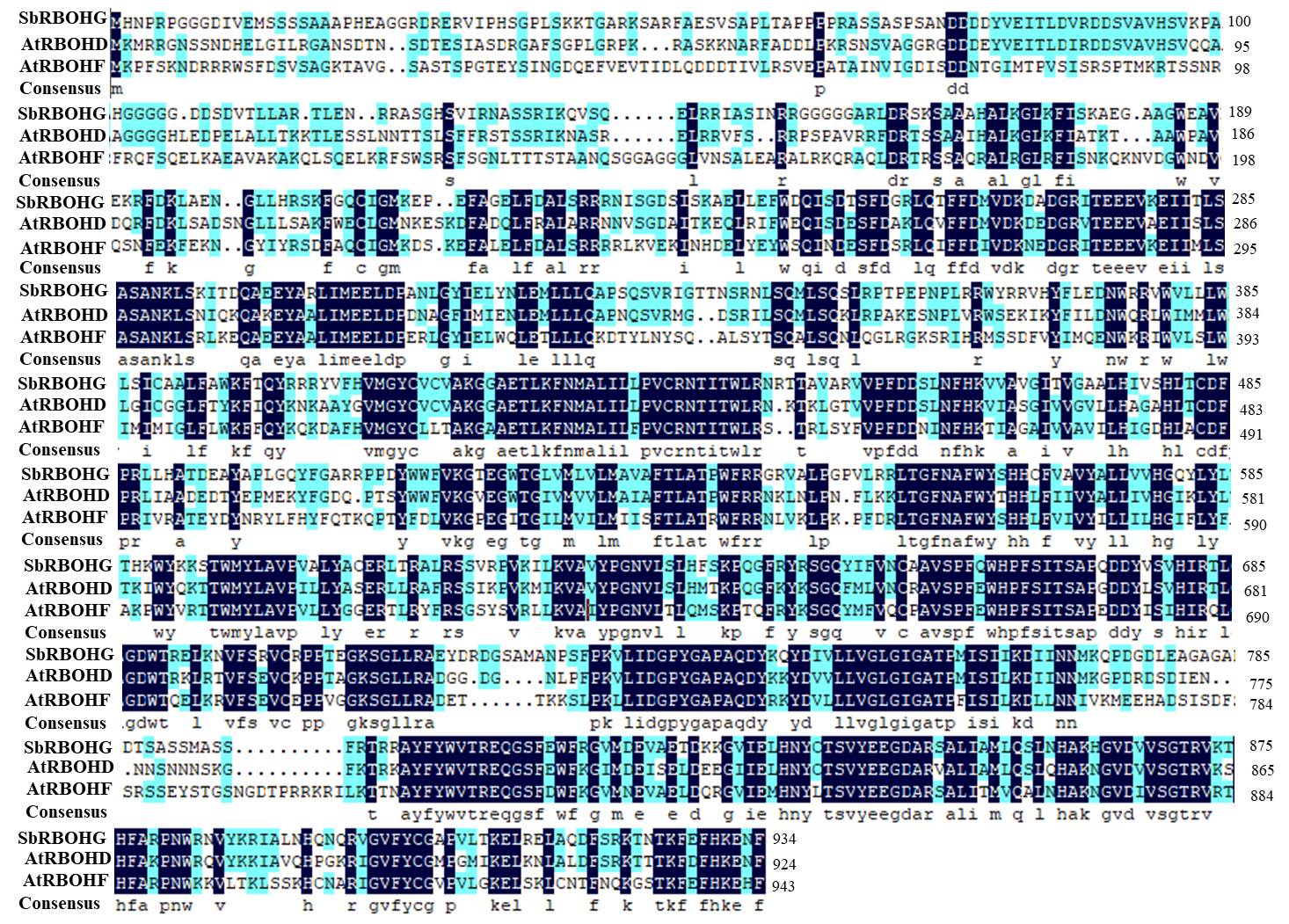


**Figure S4** Amino acid sequence alignment analysis of sorghum SbRBOHG and its homologous proteins AtRBOHD and AtRBOHF in *Arabidopsis*. Multiple sequence alignment of sorghum SbRBOHG, Arabidopsis AtRBOHD, and AtRBOHF was performed using DNAMAN software. Results indicate an overall sequence identity of 64.79% among the three proteins. Sequences are arranged top-to-bottom in the figure, with fully conserved residues highlighted on a black background and similar residues on a cyan background.


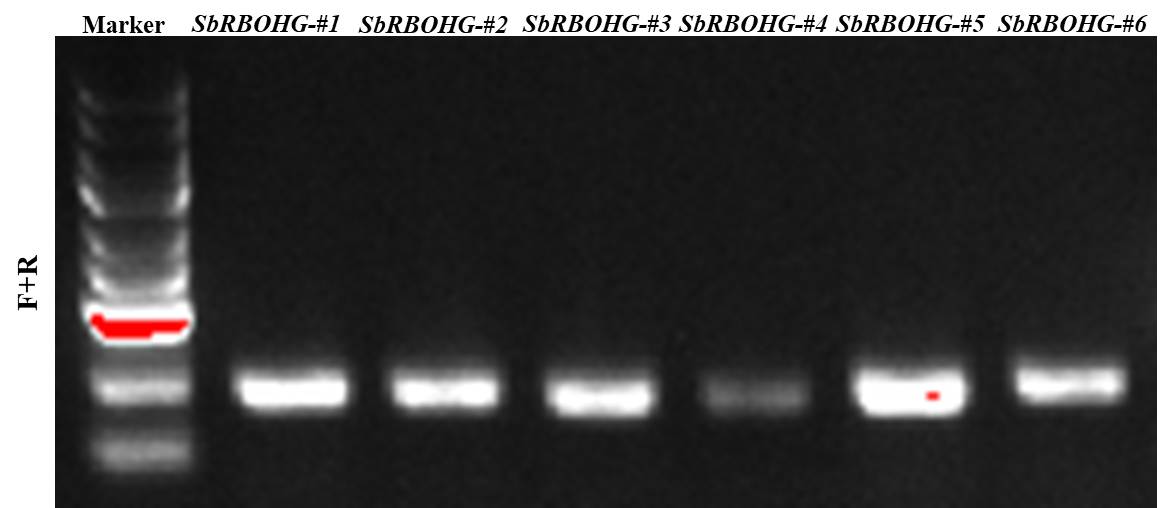


**Figure S5** RT-PCR Characterization of heterologous *SbRBOHG* overexpressing sorghum lines. RT-PCR was used to detect *SbRBOHG* gene expression in six independent transgenic Arabidopsis lines (OE-*SbRBOHG#1* to OE-*SbRBOHG#6*) obtained via *Agrobacterium*-mediated transformation. The lanes are, in order: DL2000 DNA Marker (M) and cDNA amplification products from the six transgenic lines.

**Table S1.** Primers used in this study

| **Primers** | **Sequence 5′-3′** | | **Purpose** |
| --- | --- | --- | --- |
| StActin-F | CCTGTACCGTCCCTCGACTT | RT-qPCR | |
| StActin-R | ATGCTTGCACCCTGTACTGC |  |  |
| SbRBOHA-F | CCGAAATCCCCAGCCTTGAT |  |  |
| SbRBOHA-R | CGACCGATCGACCTACTGGA |  |  |
| SbRBOHB-F | TACATGAACTACAGCCGCCC |  |  |
| SbRBOHB-R | TCCTCCTCCACCTCCTGGTA |  |  |
| SbRBOHC-F | ACATTCGCACAAGGGGTGAT |  |  |
| SbRBOHC-R | GTTTTGGGAACCTGGCGTTG |  |  |
| SbRBOHD-F | TGGAACCTACGCCTCTCTGA |  |  |
| SbRBOHD-R | GGTCTCCAGCTGCCAAATCT |  |  |
| SbRBOHE-F | GTCCAGCAGTGTCACCCTTT |  |  |
| SbRBOHE-R | GGCCACTTTTACCACCCACT |  |  |
| SbRBOHF-F | CACGGCTCTGAGATAGGCAG |  |  |
| SbRBOHF-R | TGACCTTGCATCGCCTTCTT |  |  |
| SbRBOHG-F | AACTGGCGCAACGTCTACA |  |  |
| SbRBOHG-R | GGAAAATGGCGTCTGAATGC |  |  |
| SbRBOHH-F | GGCGCGGCTCCTGAAC |  |  |
| SbRBOHH-R | CGAACTCCTTGGAGTCCACC |  |  |
| SbRBOHI-F | CATGGCGTCTGCCTCTACAT |  |  |
| SbRBOHI-R | GCTCTTGTAGCTGAACCCGT |  |  |
| SbRBOHJ-F | GCAGATGGCCATATCACGGA |  |  |
| SbRBOHJ-R | GGCCTGGCTGTAGTTCACAT |  |  |
| SbBIK1-F | GCCAACAAGTGCCAAGACTG |  |  |
| SbBIK1-R | GCCTGCAAGGCGATAATGTG |  |  |
| SbALMT1-F | ATCCCAAGCCAACGTTCACA |  |  |
| SbALMT1-R | GGTTTTTGAAGCGGGCAAGT |  |  |
| SbSTOP1-F | GAGAAGCACTGTGGTCGTGA |  |  |
| SbSTOP1-R | AGGTGTGTGGCCTTGGAAAA |  |  |
| SbRBOHG-F2 | GCGCGTGATCCCACACAG | Vector construction | |
| SbRBOHG-R2 | TAGCTAAATGCATGTGTTGATGG |  |  |
| SbBIK1-F2 | GAACTGCTGCTGCTGGGG |  |  |
| SbBIK1-R2 | GAATGGGCCAATGGTTTTGGA |  |  |
| SbRBOHG-F3 | CAACGTCTACAAGCGCATCG | Identification of overexpressing plants | |
| SbRBOHG-R3 | ACGTAGGAAAATGGCGTCTGA |  |  |

**Table S2** The naming and molecular characteristics of *SbRBOH* gene family members in sorghum.

| Gene ID | Gene name | N-amino acids | Molecular weight | Theoretical pI | Transmembrane domain | Subcellular localization |
| --- | --- | --- | --- | --- | --- | --- |
| Sb01G0039480 | *SbRBOHA* | 717 | 81032.30 | 9.58 | 6 | Plas |
| Sb02G0031550 | *SbRBOHB* | 1010 | 112247.98 | 9.11 | 6 | Plas |
| Sb03G0020050 | *SbRBOHC* | 896 | 100971.92 | 9.35 | 6 | Plas |
| Sb03G0023940 | *SbRBOHD* | 827 | 93653.13 | 9.38 | 6 | Plas |
| Sb03G0038800 | *SbRBOHE* | 944 | 106568.23 | 9.29 | 6 | Plas |
| Sb03G0045610 | *SbRBOHF* | 854 | 96516.63 | 9.42 | 6 | Plas |
| Sb05G0024410 | *SbRBOHG* | 934 | 104790.64 | 9.39 | 6 | Plas |
| Sb07G0023460 | *SbRBOHH* | 1009 | 113135.40 | 9.60 | 6 | Plas/ E.R. |
| Sb08G0020170 | *SbRBOHI* | 916 | 101764.19 | 8.94 | 6 | Plas |
| Sb09G0028620 | *SbRBOHJ* | 957 | 107460.51 | 9.43 | 6 | Plas |
